# The scientific rationale and study protocol for the DPP3, Angiotensin II, and Renin Kinetics in Sepsis (DARK-Sepsis) randomized controlled trial: serum biomarkers to predict response to angiotensin II versus standard-of-care vasopressor therapy in the treatment of septic shock

**DOI:** 10.1186/s13063-024-07995-0

**Published:** 2024-03-12

**Authors:** J. Pedro Teixeira, David Perez Ingles, Jordan B. Barton, James T. Dean, Pablo Garcia, Susan J. Kunkel, Preeyaporn Sarangarm, Natalie K. Weiss, Christopher L. Schaich, Laurence W. Busse, Nathan D. Nielsen

**Affiliations:** 1 Division of Nephrology, Department of Internal Medicine, University of New Mexico School of Medicine, Albuquerque, NM USA; 2 Division of Pulmonary, Critical Care, and Sleep Medicine, Department of Internal Medicine, University of New Mexico School of Medicine, Albuquerque, NM USA; 3 Investigational Drug Services Pharmacy, University of New Mexico Health Sciences Center, Albuquerque, NM USA; 4 Department of Pharmacy, University of New Mexico Hospital, Albuquerque, NM USA; 5 Clinical Trials Unit, Department of Internal Medicine, University of New Mexico School of Medicine, Albuquerque, NM USA; 6 Hypertension and Vascular Research Center, Wake Forest University School of Medicine, Winston-Salem, NC USA; 7 Division of Pulmonary, Allergy, Critical Care, and Sleep Medicine, Department of Medicine, Emory University School of Medicine, Atlanta, GA USA; 8 Section of Transfusion Medicine and Therapeutic Pathology, Department of Pathology, University of New Mexico School of Medicine, Albuquerque, NM USA

**Keywords:** Septic shock, Angiotensin II, Vasopressor, Renin, Biomarker, Dipeptidyl-peptidase 3, DPP3

## Abstract

**Background:**

Data to support the use of specific vasopressors in septic shock are limited. Since angiotensin II (AT2) was approved by the Food and Drug Administration in 2017, multiple mechanistically distinct vasopressors are available to treat septic shock, but minimal data exist regarding which patients are most likely to benefit from each agent. Renin and dipeptidyl peptidase 3 (DPP3) are components of the renin–angiotensin–aldosterone system which have been shown to outperform lactate in predicting sepsis prognosis, and preliminary data suggest they could prove useful as biomarkers to guide AT2 use in septic shock.

**Methods:**

The DARK-Sepsis trial is an investigator-initiated industry-funded, open-label, single-center randomized controlled trial of the use of AT2 versus standard of care (SOC) vasopressor therapy in patients admitted to the intensive care unit (ICU) with vasodilatory shock requiring norepinephrine ≥ 0.1 mcg/kg/min. In both groups, a series of renin and DPP3 levels will be obtained over the first 24 h of treatment with AT2 or SOC. The primary study outcome will be the ability of these biomarkers to predict response to vasopressor therapy, as measured by change in total norepinephrine equivalent dose of vasopressors at 3 h post-drug initiation or the equivalent timepoint in the SOC arm. To determine if the ability to predict vasopressor response is specific to AT2 therapy, the primary analysis will be the ability of baseline renin and DPP3 levels to predict vasopressor response adjusted for treatment arm (AT2 versus control) and Sequential Organ Failure Assessment (SOFA) scores. Secondary outcomes will include rates of acute kidney injury, need for mechanical ventilation and kidney replacement therapy, lengths of stay in the ICU and hospital, ICU and hospital mortality, and rates of prespecified adverse events.

**Discussion:**

With an armamentarium of mechanistically distinct vasopressor agents now available, sub-phenotyping patients using biomarkers has the potential to improve septic shock outcomes by enabling treatment of the correct patient with the correct vasopressor at the correct time. However, this approach requires validation in a large definitive multicenter trial. The data generated through the DARK-Sepsis study will prove crucial to the optimal design and patient enrichment of such a pivotal trial.

**Trial registration:**

ClinicalTrials.gov NCT05824767. Registered on April 24, 2023.

**Supplementary Information:**

The online version contains supplementary material available at 10.1186/s13063-024-07995-0.

## Background

Norepinephrine and vasopressin are currently the only two vasoconstrictive agents recommended for use in septic shock by the 2021 Surviving Sepsis Campaign Guidelines with at least moderate quality of evidence [1]. Notably, mortality data supporting their use are limited, with the mortality benefit of norepinephrine being evident primarily in meta-analyses rather than individual trials and no mortality benefit being evident in either individual trials or meta-analyses of vasopressin [2, 3]. Given that none of the currently recommended vasopressor agents used to treat septic shock have compelling outcomes data to support their use, additional studies are needed.

Angiotensin II (AT2) was approved by the U.S. Food and Drug Administration (FDA) in 2017 for the treatment of septic or other distributive shock after the Angiotensin II for the Treatment of High-Output Shock (ATHOS-3) trial [4]. In the randomized placebo-controlled ATHOS-3 trial, AT2 was found to effectively increase blood pressure and reduce the need for other vasopressor agents (with 97% of trial subjects on norepinephrine and 67% on vasopressin at randomization) without a significant increase in adverse effects. Though the trial was not powered for mortality, a non-significant trend towards decreased mortality was observed in the AT2 arm, a result which was statistically significant in a subgroup of the sickest patients [i.e., those with Acute Physiology and Chronic Health Evaluation (APACHE) II scores > 30] [5]. Despite these promising results, additional data are needed to determine which patients are most likely to benefit from AT2 therapy.

Post hoc subgroup analyses of ATHOS-3 data suggest that AT2 may be especially beneficial in patients with acute kidney injury (AKI) or acute respiratory distress syndrome (ARDS). Among subjects with AKI requiring kidney replacement therapy (KRT) at randomization, AT2 produced a statistically significant benefit in mortality and in the rate of AKI resolution [6], an effect felt to reflect its ability to increase or preserve glomerular filtration through preferential vasoconstriction of the efferent renal arteriole. Notably, though a similar effect of vasopressin has been proposed, a renal benefit was not confirmed when evaluated as the primary endpoint a large randomized controlled trial (RCT) [7]. Similarly, a non-significant trend towards decreased mortality with AT2 was observed among patients with ARDS in ATHOS-3 [8]. Such a benefit in ARDS is biologically plausible given that angiotensin-converting enzyme (ACE) is present at high levels in the pulmonary vascular endothelium, and therefore ARDS patients with septic shock may be particularly deficient in AT2. Furthermore, subsequent retrospective observational data suggest that AT2 initiation in patients with shock is associated with improvements in oxygenation, possibly through improved ventilation-perfusion matching [9].

Furthermore, AT2 use may be particularly effective in septic shock accompanied by elevated renin levels. The endothelial injury associated with septic shock has been proposed to disrupt the function of endothelial membrane-bound ACE, resulting in a state of AT2 deficiency and, through loss of negative feedback, elevated renin levels [10]. In an analysis of 20 mixed intensive care unit (ICU) patients, renin levels outperformed lactate as a prognostic tool, predicting ICU mortality with an area under the receiver operator curve of 0.80 [11]. In at least four additional prospective studies, renin levels or changes in renin levels have successfully predicted outcomes of critical illness, including AKI and AKI requiring KRT in septic shock, AKI after cardiac surgery, death and major adverse kidney events in a mixed ICU population, and mortality in hypotensive ICU patients [12–15]. In another post hoc subgroup analysis of ATHOS-3 data, AT2 use was associated with a statistically significant 19% reduction in 28-day mortality in subjects with renin concentrations above the median [10]. Renin may also serve as a gauge of response to AT2 therapy. A recent retrospective analysis of 40 patients undergoing cardiac surgery demonstrated that the use of AT2 (but not norepinephrine alone) resulted in lower subsequent renin levels [16].

Another candidate biomarker to guide the use of AT2 is dipeptidyl peptidase 3 (DPP3), an aminopeptidase that cleaves a variety of biologically active oligopeptides including AT2 [17–19]. In animal models, DPP3 modulates the renin-angiotensin system by cleaving AT2 without acting on angiotensin I (AT1), leading to an elevated AT1/AT2 ratio [20]. Elevated AT1/AT2 ratios were observed in patients with catecholamine-resistant septic shock in ATHOS-3, and, like elevated renin, an elevated AT1/AT2 ratio may identify patients likely to benefit from AT2 therapy [21]. Likewise, DPP3 levels have been found to be elevated in animal models of septic shock [22] and in human patients with sepsis, especially those with high severity of illness or those who ultimately die [23]. In a recent multicenter observational study of 600 patients with sepsis, DPP3 levels were associated with development of kidney or liver dysfunction and the need for KRT, mechanical ventilation, or vasopressor support [24]. Moreover, DPP3 levels independently predicted short-term mortality, outperforming lactate. Conversely, improved DPP3 levels were associated with improving organ function and lower risk of death. Notably, in animal models of sepsis, DPP3 inhibition improves hemodynamics and survival by mitigating septic cardiomyopathy, an effect potentially mediated by the resulting increase in AT2 levels [22]. Finally, though additional human data are needed to evaluate this theory, the use of pharmacologic AT2 has been suggested as an option to combat the hemodynamic deterioration induced by elevated DPP3 in sepsis [25].

Collectively, these data suggest that renin and DPP3 warrant additional prospective study as biomarkers in sepsis and specifically as biomarkers that may identify patients likely to benefit from AT2 therapy. However, many questions remain. As renin and DPP3 levels may serve as predictors of outcome of septic shock or critical illness in general, the relationship between their levels and responses to AT2 therapy remains unclear. Specifically, given (1) that renin and DPP3 levels are strongly associated with increased risk of death or adverse events in critical illness and (2) that AT2 may be particularly effective in patients with high severity of illness (e.g., APACHE II score > 30 in ATHOS-3 [5]), it is unclear whether renin or DPP3 levels independently predict response to AT2 or if the relationships between renin and DPP3 levels and AT2 are confounded by the underlying severity of illness. Notably, to date, all data linking renin levels to AT2 responses have been retrospective. Likewise, the ability of elevated DPP3 levels to identify patients that may benefit from AT2 has been proposed based on biologic rationale but has not yet been proven. Furthermore, as several studies [14–16, 24] found relationships between *changes* in renin or DPP3 levels and outcomes or AT2 response, it is unclear if baseline biomarker levels or changes in these levels are more useful in septic shock. Finally, little is known about the effect of stopping AT2 therapy on renin or DPP3 levels. Since elevated renin predicts poor outcome in septic shock and AT2 therapy appears to benefit patients with elevated renin and produces a decrease in renin levels, it is plausible that renin levels may rebound after AT2 therapy is stopped. However, such a rebound has never been demonstrated. Further, should a rebound effect exist, it is unknown whether this rebound carries any prognostic significance. In the case of DPP3, the kinetics of circulating DPP3 levels with either starting or stopping AT2 remain unknown.

## Study aims and objectives

This RCT will evaluate the use of renin and DPP3 levels to predict response to AT2 therapy. Hereafter, the term “biomarker” will be used to refer to levels of both renin and DPP3. We aim to prospectively clarify the relationship between biomarker blood levels, clinical responses to AT2, and severity of illness in patients with septic shock. To determine if the ability of biomarker levels to predict clinical response to vasopressor therapy is specific to AT2, we will assess biomarker levels in patients with septic shock treated with AT2 and in a control group not treated with AT2. Because we hypothesize that biomarker levels will identify patients that specifically benefit from AT2 and because the need for high-dose catecholamine monotherapy in septic shock is consistently associated with mortality [26], we will introduce AT2 therapy earlier in the course of illness than in ATHOS-3, an approach that appears safe based on recent pilot trial data [27] and potentially beneficial based on additional post hoc analyses of ATHOS-3 data [28]. To shed light on the relationship between AT2 response, overall outcome, biomarker levels, and changes in biomarker levels, we will collect biomarker levels at multiple time points in patients treated with AT2 and controls. Finally, to investigate if biomarker rebound occurs after AT2 discontinuation and if such a rebound carries any prognostic significance, we will obtain biomarker levels after AT2 discontinuation in the AT2 arm.

## Methods and analysis

### Design

**Fig. 1:**
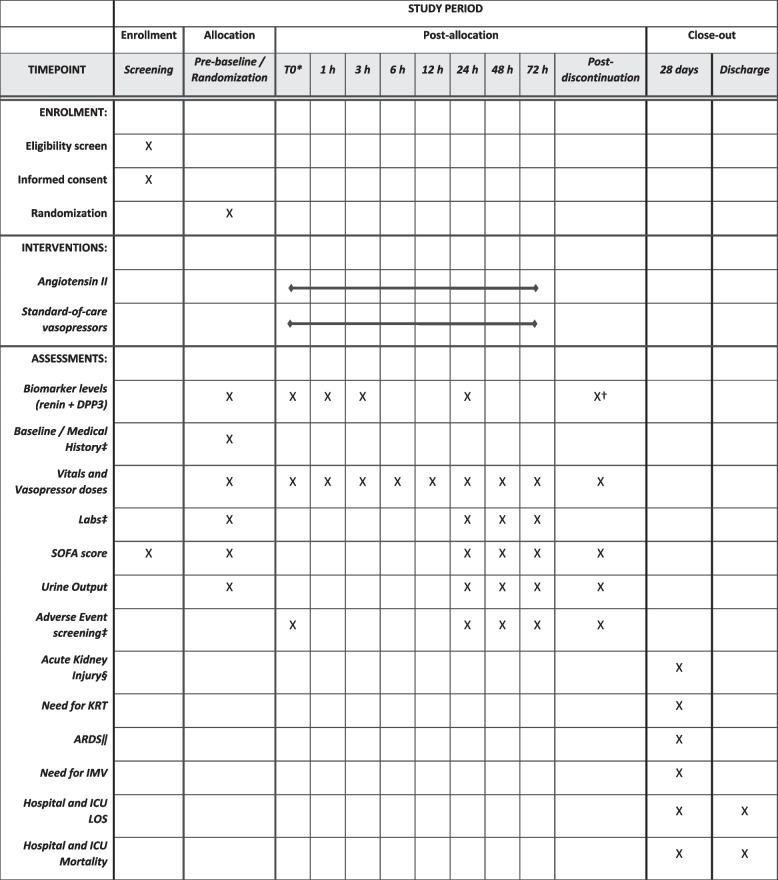
Schedule of enrollment, interventions, and assessments. *Time zero is defined as 2 h after randomization in the standard of care arm and as time of angiotensin II initiation in the intervention arm. †Post-discontinuation biomarker levels will be obtained only in the angiotensin II arm. ‡See Data Collection Form for a full list of baseline characteristics/comorbidities/medical history, laboratory values, and adverse events being collected. §Acute kidney injury will be defined and staged using creatinine-based 2012 KDIGO (Kidney Disease Improving Global Outcomes) criteria [30]. ‖Diagnosis of ARDS will be ascertained via screening of clinical notes and corroborated by the investigators using the 2012 Berlin Criteria [31]. Abbreviations: DPP3, dipeptidyl-peptidase 3; ICU, intensive care unit; IMV, invasive mechanical ventilation; LOS, length of stay; KRT, kidney replacement therapy; SOFA, sequential organ failure assessment

This is an investigator-initiated industry-funded single-center RCT. As we are comparing the addition of AT2 to a control group treated with standard-of-care (SOC) vasopressors and the primary outcome includes quantification of total vasopressor dose (including the component of AT2 dose in the AT2 arm), both the trial execution and the analysis will be unblinded. This trial protocol was designed using the elements of the Standard Protocol Items: Recommendations for Interventional Trials (SPIRIT) 2013 Checklist [29]. The checklist is included as supplementary material 1 and includes additional information on the specific trial team members responsible for trial oversight and coordination, endpoint adjudication, data management, and safety monitoring. See Fig. 1 for the schedule of study activities. As this is a mechanistic study with a physiologic rather than clinical primary endpoint, neither patients nor public were involved in the study design.

### Setting

The study will be carried out in the adult ICUs at the University of New Mexico Hospital (UNMH). UNMH is a publicly funded, tertiary care center serving as the primary safety net hospital for the state of New Mexico, providing care to a medically underserved population [32]. The adult ICUs at UNMH have 72 beds cared for by medical, trauma-surgical, neurosciences, and cardiothoracic-vascular ICU services.

### Screening, eligibility, and consent

Patients with septic or other vasodilatory shock admitted to the ICU from the emergency department or ward will be identified by discussion with the ICU providers and screening the electronic medical record (EMR) for all ICU patients admitted to the hospital within the prior 96 h, including patients directly admitted to the ICU and those admitted to the wards and subsequently transferred to the ICU. All subjects receiving vasopressor therapy within the prior 48 h that do not meet inclusion criteria will be considered screened but excluded; their medical record numbers, date of screening, and reason(s) for exclusion will be recorded in a screening database maintained by the research coordinators and stored on a secured UNM Health Sciences Center (HSC) network drive.

Inclusion and exclusion criteria are outlined in Table 1. Notably, the diagnosis of vasodilatory shock in our study is not protocolized but is rather a clinical diagnosis made by treating clinicians. For patients with impaired mental status unable to provide informed consent, the legal authorized representative (LAR) will be approached for consent. See Supplementary Material 2 for the full consent form. Subjects or their LARs may request withdrawal from the study at any point for any reason.

**Table 1:** Study inclusion and exclusion criteria

Inclusion criteria	Exclusion criteria
• Adults patients ≥ 18 years old with persistent vasodilatory shock despite moderate-dose vasopressor therapy, defined as those who require norepinephrine or other vasopressor therapy with total norepinephrine equivalent dose (NED) at ≥ 0.1 mcg/kg/min for at least 30 min to maintain a MAP between 65 and 70 mm Hg [4, 33, 34] • Central venous and arterial catheters present and expected to remain in place for ≥ 72 h • Patients must have received ≥ 20 mL/kg of crystalloid over the previous 24-h period, as clinically appropriate, and no longer be fluid responsive as per local protocol^a^. Patients for whom the treating physicians feel that 20 mL/kg of crystalloid may be clinically inappropriate may qualify for the study if the reason for withholding further IV fluids is documented • Patient or legal authorized representative is willing and able to provide written informed consent and comply with all protocol requirements • Approval from the attending physician supervising care of the patient and the clinical pharmacist conducting the study	• Age < 18 years • Acute occlusive coronary syndrome requiring intervention and/or cardiogenic shock • Known or suspected abdominal aortic aneurysm or aortic dissection • Acute stroke • Acute mesenteric ischemia or history of mesenteric ischemia • Known Raynaud’s phenomenon, systemic sclerosis, or vasospastic disease • Requirement for venoarterial ECMO • Liver failure with a MELD score [35] of ≥ 30 • Burns covering > 20% of total body surface area • History of asthma or COPD with active acute bronchospasm or (if not mechanically ventilated) with an acute exacerbation of their asthma/COPD requiring the use of inhaled bronchodilators • Requirement for > 500 mg daily of hydrocortisone or equivalent glucocorticoid medication as a standing dose • Absolute neutrophil count < 1000/mm^3^ • Hemorrhagic shock OR active bleeding AND an anticipated need (within 48 h of initiation of the study) for transfusion of > 4 units of packed red blood cells • Active bleeding AND hemoglobin < 7 g/dL or any other condition that would contraindicate serial blood sampling • Untreated venous thromboembolism (VTE) or inability to tolerate pharmacologic VTE prophylaxis • Known allergy to mannitol • Expected survival < 24 h, SOFA score [36] ≥ 16, or death deemed to be imminent or inevitable during the admission • Comfort measures status (limitations such as DNR or DNI status permitted if the patient is otherwise pursuing life-prolonging therapies) • Pregnancy (all women ≤ 50 years-old require negative serum quantitative beta-hCG to enroll) • Prisoner status • Current participation in another interventional clinical trial

^a^In our local sepsis protocol, lack of fluid responsiveness is considered a failure to increase stroke volume, stroke volume index, cardiac output, or cardiac index, usually measured by non-calibrated pulse contour analysis with a FloTrac device (Edwards Lifesciences, Irvine, CA, USA), by at least 10% after a 500-mL crystalloid bolus or a passive leg raise. Additional abbreviations: *COPD* chronic obstructive pulmonary disease, *DNI* do-not-intubate, *DNR* do-not-resuscitate, *ECMO* extracorporeal membrane oxygenation, *MAP* mean arterial pressure, *MELD* Model for End-Stage Liver Disease, *SOFA* Sequential Organ Failure Assessment

### Intervention and study procedures

The intervention will be randomization, with 1:1 allocation, to the use of AT2 or to continued use of SOC vasopressors, in both cases followed by serial determination of serum biomarker levels.

In the intervention arm, AT2 will be initiated at 20 ng/kg/min. Thereafter, in both arms, AT2, norepinephrine, and/or other vasopressors will be titrated according to the schema in Table 2, in concordance with the UNMH Nursing Department Titration Guideline (see Supplementary Material 3). In both study arms, a mean arterial pressure (MAP) goal of ≥ 65 mm Hg will be required during the first 72 h of the study period. This MAP goal will be the only aspect of care that will be dictated by study protocol in the control arm. In the AT2 arm, if additional vasopressor(s) are required to maintain MAP goal, the clinical ICU team will be responsible for selecting and ordering those agent(s). At 72 h, the patients in the AT2 arm that continue to require AT2 will have AT2 weaned off, with norepinephrine increased as needed. Subjects requiring > 0.2 mcg/kg/min of norepinephrine to wean off AT2 will have alternative SOC vasopressor agent(s), as selected by the treating ICU team, initiated. The ICU RN will have 1–2 h (i.e., until hour 73–74) to complete the AT2 wean. In both arms, the MAP goal after 72 h will be dictated by the treating ICU team rather than the research team.

**Table 2:** Angiotensin II titration scheme for angiotensin II arm

**Norepinephrine** (mcg/kg/min)	**Angiotensin II** (ng/kg/min)
< 0.05	5^a^
0.05–0.1	10
0.11–0.20	20^b^
0.21–0.25	25
0.26–0.30	30
0.31–0.35	35
> 0.35	40 (max)

^a^Angiotensin II doses of 2.5 and 1.25 ng/kg/min can be used when vasopressors are being weaned. ^b^The starting dose of angiotensin II per the U.S. Food and Drug Administration Prescribing Information is 20 ng/kg/min. When vasopressors are being weaned within these displayed ranges, norepinephrine should be weaned before angiotensin II

In both arms, serial blood draws will be obtained at time points as outlined in the biomarker assay schedule in Table 3, with an additional level obtained 24 h after AT2 discontinuation in the AT2 arm. For renin testing, 2-mL samples of blood will be collected (preferentially from an arterial catheter, whenever feasible) in an ethylenediaminetetraacetic acid (EDTA, lavender top) tube. Subjects will remain recumbent for at least 30 min prior to each renin assay. The samples will be transported to the research laboratory, centrifuged, and processed for storage at − 80 °C. The samples will later be thawed to perform the renin assays in batches of ≥ 30 assays at a time. Renin assays will be performed using the Human Renin Quantikine ELISA Kit (R&D Catalog # DREN00, R&D Systems, Inc., Minneapolis, MN). Though plasma renin activity (PRA), rather than concentration, has traditionally been used in clinical care, renin concentration assays have been developed and validated for clinical use and are now considered equivalent to PRA for the diagnosis of primary hyperaldosteronism [37, 38]. Notably, the above studies analyzing the significance of renin in critical illness all measured renin concentrations. Furthermore, the recent analysis of the ATHOS-3 cohort which demonstrated that AT2 use was associated with improved survival in patients with elevated renin levels used a similar enzyme-linked immunosorbent assay (ELISA), supporting the applicability to critical illness of renin as specifically measured by ELISA [10]. For DPP3 testing, 1-mL samples of whole blood will be collected (preferentially from an arterial catheter, whenever feasible) in a lavender top (EDTA) tube, allowed to reach room temperature and promptly analyzed using a point-of-care luminometric immunoassay (Sphingotest IB10 DPP3, 4TEEN4 Pharmaceuticals GmbH/SphingoTec GmbH, Hennigsdorf, Germany). Though not yet in clinical use, measurement of DPP3 using luminometric immunoassay has been validated in both critically ill patients and healthy controls [23]. All blood samples will be discarded after the trial.

**Table 3:** Biomarker assay schedule

Time point	Angiotensin II arm	Control (SOC) arm
Pre-baseline	Randomization	Randomization
Baseline (T0)	Drug initiation	2 h post-randomization
1 h	1 h post-drug initiation^a^	3 h post-randomization^a^
3 h	3 h post-drug initiation^a^	5 h post-randomization^a^
24 h	24 h post-initiation^b^	26 h post-randomization^b^
Post-discontinuation	24 h post-drug discontinuation^c^	N/A

SOC schedule built on estimation that time from randomization to study drug initiation will be approximately 2 h. ^a^ ± 15 min. ^b^ ± 30 min. ^c^ ± 8 h. Abbreviations: *SOC* standard of care

Apart from the biomarker assays, all data collected will be prospectively sourced from the EMR, as these measures are routinely obtained as part of SOC in the ICU. In addition, as the study drug will be delivered by ICU nurses and all biomarker samples will be obtained by the research team, the trial study design will inherently assure protocol adherence (barring withdrawal of consent). The requirement for arterial and central venous catheter placement as an inclusion criterion will eliminate pain associated with blood sampling and thereby enhance subject retention.

### Safety monitoring

During the treatment phase over the initial 96 h of the study, the patients will be screened at least daily for adverse events (AEs). As many of the potential AEs are common complications of shock or critical illness, AEs will include only new hospital-acquired events that develop after randomization. A series of prespecified AEs will be tracked (see Table 4), which include a variety of known potential complications of vasopressor use and adverse reactions listed in the FDA Prescribing Information for AT2. The latter consist of AEs that occurred with AT2 in ATHOS-3 at a rate of ≥ 4% and ≥ 1.5% more than in the placebo group. At any point, the investigators or the ICU providers may request study termination for safety reasons.

**Table 4:** Secondary outcomes

Key secondary clinical outcomes: • Days free from KRT (up to 28 days) • Days free from mechanical ventilation (up to 28 days) • ICU mortality • Hospital mortality
Additional secondary clinical outcomes: • Vasopressor response, as quantified by decrease in NED of background vasopressor(s) in the AT2 arm and total vasopressor requirement in NED in the control arm (i.e., primary proximate endpoint), will be re-assessed at multiple additional time points: o 1 h o 6 h o 12 h o 24 h o 48 h o 72 h • Time to sustained shock reversal (as defined by vasopressor independence) • Change in SOFA [36] scores and organ-specific SOFA sub-scores at 24 h, 48 h, and 72 h • Frequency of acute kidney injury (as defined by KDIGO criteria [30]) • ICU length of stay • Hospital length of stay
Exploratory analyses of biomarker kinetics: • Repeating the primary analysis (i.e., the ability of baseline biomarker levels to predict change in total NED at 3 h, adjusted for baseline SOFA score and treatment arm) using *pre-baseline* biomarker levels obtained immediately upon patient entry into the study (instead of baseline biomarker levels, obtained with initiation of AT2 therapy in the AT2 arm or 2 h after randomization in the control arm) • Correlations between *changes* in biomarker levels and change in total NED or ICU mortality • In the AT2 arm, we will assess for presence of rebound effect by measuring biomarker levels 24 (± 8) h post-drug discontinuation and, if present, will assess for correlation of rebound effect with ICU mortality
Prespecified safety endpoints/adverse events (AEs):^a^ • New venous thromboembolism or arterial thrombosis diagnosed during hospital stay • Atrial fibrillation • Tachycardia (heart rate > 100/min sustained for ≥ 1 h) • Lactic acidosis • Peripheral limb/digital ischemia • Intestinal ischemia • Thrombocytopenia • Hyperglycemia • Confirmed infection (with infecting organism confirmed by culture or other identification method; administration of appropriate antimicrobial therapy; and clinical documentation of infection) • Any other potentially related AEs will be recorded

^a^For these to be considered AEs, they must be new hospital-acquired events which developed after randomization. Additional Abbreviations: *AT2* angiotensin II, *ICU* intensive care unit, *KRT* kidney replacement therapy, *NED* norepinephrine equivalent dose, *SOFA* Sequential Organ Failure Assessment

### Outcome measures and analysis plan

#### Primary outcome

The primary outcome is an as-treated analysis of whether baseline biomarker levels are useful in predicting the response to AT2 when added to moderate-dose vasopressor therapy. To assess this primary outcome, we will define a *primary proximate endpoint* as response to vasopressor therapy — defined as a reduction in dose of vasopressor therapy as measured by norepinephrine equivalent dose (NED) [33, 34] — at 3 h post-drug initiation in the AT2 arm and the equivalent time point in the control arm. NED (in mcg/kg/min) will be computed using the equivalency equation published by Kotani et al. in 2023 which includes AT2 as follows: norepinephrine dose (mcg/kg/min) + epinephrine dose (mcg/kg/min) + 0.01 × dopamine dose (mcg/kg/min) + 0.06 × phenylephrine dose (mcg/kg/min) + 2.5 × vasopressin dose (U/min) + 0.0025 × angiotensin II dose (ng/kg/min). We will consider the levels of our two biomarkers at baseline (T0; see Table 3) our *major independent variables*. To assess the influence of the severity of illness on the relationship between baseline biomarker levels and vasopressor response, Sequential Organ Failure Assessment (SOFA) [36] scores will be tabulated at baseline (T0) and will be considered our *primary covariable*. Our analysis will be carried out in two steps. First, to evaluate the ability of baseline biomarker levels to predict response to AT2 therapy, we will perform two univariable regression analyses between each biomarker level and change in *background* NED at 3 h within the AT2 arm. Second, to determine if the ability of the biomarkers to predict vasopressor response is specific to AT2 therapy, in our *primary analyses* we will perform two multivariable analyses (one for each biomarker), with the biomarker levels the independent variable, the change in *total* NED at 3 h the dependent variable, with SOFA score and treatment arm (AT2 versus control) as covariables.

Given the possibility that the product of the biomarkers will outperform each individual biomarker [39], we will perform a sensitivity analysis using the product of the biomarkers as the independent variable. Likewise, given the lack of prior data describing the relationship between biomarker levels and NED, whether this relationship is linear is unknown. As such, in addition to the primary analysis assuming a linear relationship, we will perform sensitivity analyses for non-linear relationships. First, we will perform three analyses in which we log transform the baseline biomarker levels, change in NED, and both. Second, though the statistical power of such analyses will be limited, we will directly assess for curvilinear relationships by fitting splines and comparing nested models with first-order and higher order terms (e.g., quadratic, cubic) via likelihood ratio test for goodness of fit.

Results will be presented as confidence intervals with 95% confidence intervals.

#### Secondary and exploratory outcomes

A variety of secondary outcomes will be evaluated (see Table 4 for a full listing). Key secondary outcomes include days free from KRT at day 28, days free from mechanical ventilation at day 28, ICU mortality, and hospital mortality.

We will perform a series of exploratory analyses evaluating the significance of renin and DPP3 kinetics. Specifically, we will repeat the primary multivariable analysis using pre-baseline biomarker levels in place of baseline levels; we will analyze whether changes in biomarker levels (e.g., between baseline and 1 h, 3 h, or 24 h) predict response to vasopressor therapy (i.e., change in total NED requirement) or overall outcome (i.e., ICU mortality); and assess for the presence of a rebound effect using the post-discontinuation biomarker levels and, if present, assess whether the rebound correlates with outcome (i.e., ICU mortality).

Finally, though the statistical power of such analyses will be limited, we will perform additional exploratory subgroup analyses to evaluate if patients with septic shock complicated by AKI or septic shock complicated by ARDS specifically benefit from AT2 therapy with regard to vasopressor response (i.e., change in total NED at 3 h) and ICU mortality.

### Sample size estimation

The primary analyses, one each for renin and DPP3, will consist of multivariable regression including 3 predictor variables. With 3 predictor variables, a power (1-beta) of 0.8, and significance level (alpha) of 0.05, a sample size of 36 is required to detect a moderate-to-large effect size (i.e., *f*^2^ of 0.35).

### Randomization procedure

Random group allocation in a 1:1 ratio will be performed by UNMH Investigation Drug Services (IDS) pharmacists and will be completely separated from the investigators recruiting patients for the trial. A randomization table was generated using the RAND() function in Excel (Microsoft Corporation, Redmond, WA, USA) and an appropriate block size to ensure equal but unpredictable group assignments, with the block sizes blinded to the other investigators. The randomization table will be stored securely in a location accessible only to IDS staff. Once an individual subject or LAR consents to the trial, the investigator will notify the IDS pharmacist who will access the randomization table and report the group assignment back to the investigator. In patients allocated to the AT2 arm, randomization will trigger preparation of study drug by the IDS pharmacist.

### Data collection and management

Data will be collected by two research coordinators using a local paper data collection form (DCF, see Supplementary Material 4) and a corresponding web-based secure remote data capture system (REDCap) [40]. The research coordinators will be personally trained by a co-primary investigator (co-PI) via direct supervision of the data entry for the first five subjects. Additional assurance of the quality of data acquisition will include manual audits of the data entry carried out by the study co-PI(s) for every fifth patient enrolled.

Clinical data will be collected for up to 28 days after randomization or until hospital discharge, whichever occurs first. Subject identification in both the DCF and web-based system will be through a unique study number. Detailed data will be collected daily from the time the patient is randomized through day 4 after randomization (i.e., 24 h after the 72-h intervention period is complete). Thereafter, data on new adverse events and major outcomes (e.g., discharge, death, and need for organ support) will be collected on a weekly basis until discharge. After 28 days, the only data collected will be the length of ICU and hospital stay and in-hospital mortality.

Confidentiality of the data obtained from enrolled participants will be achieved by storing the paper DCFs in secure locked cabinets in the UNM Department of Internal Medicine Clinical Trials Unit and via the use of the secure web-based REDCap data management tool. Each patient will be assigned a unique research ID, which will be used to identify the REDCap record and the biospecimens in storage for each patient. Once all data are collected, the records will be de-identified by removing any identifying information including medical record numbers, names, and dates of birth and hospital admission.

Given this is a single-center study comparing FDA-approved medications used for their approved indications, the study will not include a data monitoring committee, an interim analysis or stopping rules, or a trial-specific auditing plan. See SPIRIT Checklist Items 5, 21, and 23 (Supplementary Material 1) for additional details. Should any unforeseen circumstance develop which would potentially preclude the ongoing safe execution of the trial, the co-PIs will together decide whether to terminate the trial and this decision will be completely independent of the trial sponsor.

### Funding and material support

Funding for this investigator-initiated study and the supply of AT2 are provided by La Jolla Pharmaceutical Company (LJPC, an affiliate of Innoviva Specialty Therapeutics), the marketer and distributor of AT2. The trial protocol was independently designed by the investigators and subsequently submitted to LJPC, who reviewed and approved the protocol. All generated data will remain under the control of the investigators, and the results will be published regardless of the outcome of the trial. The sponsor will have no role in the data collection, data analysis, data interpretation, writing of the final study manuscript, or the decision to submit the report for publication.

The DPP3 analyzer (Sphingotest IB10 DPP3) and cartridges used in this study were donated to UNM by 4TEEN4 Pharmaceuticals GmbH/SphingoTec GmbH to carry out this research. Neither company had any role in the protocol design.

### Ethics and dissemination

#### Regulatory and ethical approval

The protocol was approved by UNM HSC Human Research Protections Office (protocol #22–111, initial approval April 27, 2022; most recent version 2.3 approved July 17, 2023) and is posted on ClinicalTrials.gov (NCT05824767). Any significant protocol revisions will be approved by our institutional review board, communicated to the sponsor, communicated to *Trials*, and updated on the ClinicalTrials.gov registry.

#### Dissemination policy

We intend to disseminate findings to participants, healthcare professionals, and the public via conference presentation(s), publication in peer-reviewed journal(s), and social media without any publication restrictions. The final manuscript will be drafted by the primary investigators.

#### Data access policy

The final patient-level de-identified dataset will be made accessible upon reasonable request once the results are published. Such requests will require regulatory approval by both UNM and LJPC.

## Limitations

Limitations of our study include the limited sample size and single-center design which inherently limit statistical power and generalizability. Regarding the sample size, no previously published prospective data exist about our primary outcome to inform sample size estimation. A major goal of this study is to assess the effectiveness of baseline biomarker levels to predict outcomes, thus informing the power analyses of subsequent studies. However, we felt that a robust signal (i.e., *f*^2^ of 0.35) would be required in order to justify the use of these biomarkers as enrichment tools in the inclusion criteria of future trials of AT2.

An additional limitation is the unblinded nature of the study and analysis which increases the risk of bias. However, given that the primary independent variables (i.e., biomarker levels) and primary dependent variable [i.e., dose of vasopressor(s) titrated by ICU nurses independent of the study team and targeted to a fixed MAP goal in all subjects] are entirely or largely objective datapoints, the risk of bias is somewhat mitigated.

Furthermore, though many of the study design elements of our protocol were adopted from ATHOS-3, we opted not require a protocolized approach to the diagnosis of vasodilatory shock in our study as we do not routinely measure elements of the definition used by ATHOS-3, namely central venous pressure (CVP), central venous oxygen saturation (S_cv_O_2_), or cardiac index in patients with vasodilatory shock. With the recent series of large RCTs clearly demonstrating a lack of benefit of early goal-directed therapy [41–43], our local practice patterns no longer include routine measurement of CVP and S_cv_O_2_ in patients with septic shock. Likewise, apart from the frequent use of qualitative point-of-care echocardiography, we typically only measure cardiac index in patients with a clinical diagnosis of vasodilatory shock when we have a strong suspicion of an alternative shock type. While a lack of strict definition of vasodilatory shock may lead to the inadvertent inclusion of patients with other types of shock, it also would tend to increase the generalizability of our results as they will better reflect current real-world practice.

## Discussion

Despite advances in sepsis care and gradual improvements in individual patient survival rates over the last few decades, sepsis-related deaths continue to rise as the incidence of sepsis increases [44–46]. Furthermore, the current recommended approach to vasoactive therapy in septic shock remains a rather rudimentary one-size-fits-all method [1]. As the armamentarium of vasopressors available grows to include mechanistically distinct agents, sub-phenotyping patients using biomarkers and clinical parameters has the potential to improve septic shock outcomes by enabling treatment of the correct patient with the correct vasopressor at the correct time [47].

However, such an approach, though conceptually appealing, requires validation in a large definitive multicenter RCT. But before we can design such a definitive RCT, data are needed to determine which biomarker(s) should be used for patient sub-phenotyping. In the case of AT2 therapy, renin and DPP3 are two promising candidate biomarkers that are either commercially available or under development for point-of-care use in the ICU. The DARK-Sepsis trial aims to determine if these biomarkers can predict response to AT2 with regard to vasopressor response as measured by changes in NED.

Though an intermediary outcome, the use of change in NED as an outcome has important advantages. First, vasopressor dose requirements have been consistently shown to correlate with mortality in septic shock [26]. Second, as an intermediary outcome and a continuous variable, a statistically significant change in NED is far more likely to be detected in this foundational study than a binary clinical outcome such as mortality. The detection of such a signal in DARK-Sepsis would justify the use of renin or DPP3 as biomarkers to enrich the study population of a large clinical efficacy trial of AT2 in septic shock.

Given the rapid onset and short half-life of AT2, other timepoints could have reasonably been chosen for the evaluation of the primary endpoint of change in NED, but we chose to adopt the 3-h timepoint utilized by the ATHOS-3 trial [4]. However, we plan to collect and analyze data from multiple other timepoints in our secondary analyses. Similarly, though somewhat arbitrary, we opted to only screen patients within 96 h of admission to capture the majority of patients with an initial episode of vasodilatory shock but exclude those with nosocomial septic shock which has a distinct, poorer prognosis [48–50].

Though substantial observational data on the use of AT2 in real-world practice and the significance of renin and DPP3 are accumulating, the prospective interventional nature of the DARK-Sepsis trial and the inclusion of a control arm for comparison will allow us to specifically dissect whether these biomarkers serve as general prognostic markers in sepsis or if, as we hypothesize, they can be used specifically to predict response to AT2. While the use of an RCT design for such a mechanistic study is less common, our study design — in which patients in both arms are treated with FDA-approved agents for their approved indications — allows us to generate randomized data with limited additional risk (beyond the substantial risk inherent in the treatment of septic shock with vasopressor therapy), especially considering the emerging data that AT2 is safe when used as a first-line vasopressor therapy in vasodilatory shock [27]. In addition, though randomization will mitigate between-group differences in severity of illness, adjustment for severity of illness is vital in interpreting within-group assessments of the ability of these biomarkers to predict vasopressor response. Though the primary analysis of this study will evaluate between-group differences, the ability of these biomarkers to predict vasopressor response within each group is of interest as well.

Notably, the DARK-Sepsis is neither a true pilot study, as our outcome is not a feasibility metric, nor, given the RCT design, a typical preclinical study. Rather, the DARK-Sepsis study is a translational study, harnessing an RCT design — given the clinical equipoise surrounding the choice of vasopressor therapy in septic shock — and a meaningful intermediary endpoint (vasopressor response), specifically evaluating the use of candidate biomarkers which may allow for identification of patients likely to benefit from AT2 therapy. The data generated by the DARK-Sepsis study will prove crucial in the optimal design and patient enrichment of a pivotal clinical efficacy trial of AT2 in septic shock.

## Trial status

Protocol version 2.3 (approved July 17, 2023). Recruitment began April 24, 2023. The anticipated date of recruitment completion is December 2024.

### Supplementary Information

**Additional file 1.** SPIRIT checklist.

**Additional file 2.** Informed consent form approved by UNM institutional review board.

**Additional file 3.** University of New Mexico Hospital Nursing Department Titration Guideline.

**Additional file 4.** Data collection form (DCF).

## Data Availability

The final patient-level de-identified dataset will be made accessible upon reasonable request once the results are published. Such requests will require regulatory approval by both the University of New Mexico and La Jolla Pharmaceutical Company.

## Abbreviations

ACE: Angiotensin-converting enzyme
AE: Adverse event
AKI: Acute kidney injury
APACHE II score: Acute Physiology and Chronic Health Evaluation II score
ARDS: Acute respiratory distress syndrome
ATHOS-3 trial: Angiotensin II for the Treatment of High-Output Shock trial
AT1: Angiotensin I
AT2: Angiotensin II
CVP: central venous pressure
DARK-Sepsis trial: DPP3, Angiotensin II, and Renin Kinetics in Sepsis trial
DCF: Data collection form
DPP3: Dipeptidyl-peptidase 3
EDTA: Ethylenediaminetetraacetic acid
ELISA: Enzyme-linked immunosorbent assay
EMR: Electronic medical record
FDA: Food and Drug Administration
ICU: Intensive care unit
IDS: Investigation Drug Services
KRT: Kidney replacement therapy
LAR: Legal authorized representative
LJPC: La Jolla Pharmaceutical Company
MAP: Mean arterial pressure
NED: Norepinephrine equivalent dose
PRA: Plasma renin activity
RCT: Randomized controlled trial
S_cv_O_2_: Central venous oxygen saturation
SOC: Standard of care
SOFA score: Sequential Organ Failure Assessment score
SPIRIT: Standard Protocol Items: Recommendations for Interventional Trials
UNMH: University of New Mexico Hospital
UNM HSC: University of New Mexico Health Sciences Center
