# Supplementary material for: The scientific rationale and study protocol for the DPP3, Angiotensin II, and Renin Kinetics in Sepsis (DARK-Sepsis) randomized controlled trial: serum biomarkers to predict response to angiotensin II versus standard-of-care vasopressor therapy in the treatment of septic shock
Source: Trials. 2024 Mar 12;25:182. doi: 10.1186/s13063-024-07995-0 (PMC10935947; doi:10.1186/s13063-024-07995-0)
Supplement: Supplementary file 1 — Additional file 1. SPIRIT checklist. [file 13063_2024_7995_MOESM1_ESM.pdf]

SPIRIT 2013 Checklist: Recommended items to address in a clinical trial protocol and related documents*

Section/item	Item No	Description
**Administrative information**		
Title	1	Descriptive title identifying the study design, population, interventions, and, if applicable, trial acronym: ***Page 1, lines 1-3***
Trial registration	2a	Trial identifier and registry name. If not yet registered, name of intended registry: ***Page 3, line 9-10***
	2b	All items from the World Health Organization Trial Registration Data Set: ***see attachment below***
Protocol version	3	Date and version identifier: ***Page 16, line 7***
Funding	4	Sources and types of financial, material, and other support: ***Page 15 line 18 to Page 16 line 3*** and ***Page 22, line 7-10***
Roles and responsibilities	5a	Names, affiliations, and roles of protocol contributors: ***Title Page*** and ***Page 22, lines 12-19***
	5b	Name and contact information for the trial sponsor: ***see WHO trial registration data set below or ClinicalTrials.gov posting***
	5c	Role of study sponsor and funders, if any, in study design; collection, management, analysis, and interpretation of data; writing of the report; and the decision to submit the report for publication, including whether they will have ultimate authority over any of these activities: ***Page 15 line 18 to Page 16 line 3*** and ***Page 22, line 7-10***

	5d	Composition, roles, and responsibilities of the coordinating centre, steering committee, endpoint adjudication committee, data management team, and other individuals or groups overseeing the trial, if applicable (see Item 21a for data monitoring committee): ***Given the size of our study, we do not have formal committees to address these functions, but they will be carried out by the following trial leadership as follows:***<br>***--trial coordination: JP Teixeira (co-PI), ND Nielsen (co-PI), NK Weiss (clinical trials unit senior research nurse)***<br>***--trial steering: JP Teixeira (co-PI), ND Nielsen (co-PI), NK Weiss (clinical trials unit senior research nurse), LW Busse (senior advisor / methodologist)***<br>***--endpoint adjudication: JP Teixeira (co-PI), ND Nielsen (co-PI), NK Weiss (senior clinical trial unit research nurse)***<br>***--data management team: JP Teixeira (co-PI), CL Schaich (trial statistician)***<br>***The co-PIs and senior research RN meet at least weekly to coordinate the study and discuss trial activities and communicate at least daily while any patient is enrolled in the treatment phase of the trial. The co-PIs communicate at least monthly with our external senior advisor for purposes of trial steering.***
**Introduction**		
Background and rationale	6a	Description of research question and justification for undertaking the trial, including summary of relevant studies (published and unpublished) examining benefits and harms for each intervention: ***Page 4, line 1 to Page 7, line 10***
	6b	Explanation for choice of comparators: ***Page 7, lines 16-18***
Objectives	7	Specific objectives or hypotheses: ***Page 7, line 12 to Page 3, line 3***
Trial design	8	Description of trial design including type of trial (eg, parallel group, crossover, factorial, single group), allocation ratio, and framework (eg, superiority, equivalence, noninferiority, exploratory): ***Page 8, lines 6-10; page 9, lines 16-17;*** and ***page 11, line 22 to page 12, line 16.***
**Methods: Participants, interventions, and outcomes**		
Study setting	9	Description of study settings (eg, community clinic, academic hospital) and list of countries where data will be collected. Reference to where list of study sites can be obtained: ***Page 8, lines 18-22***
Eligibility criteria	10	Inclusion and exclusion criteria for participants. If applicable, eligibility criteria for study centres and individuals who will perform the interventions (e.g., surgeons, psychotherapists): ***Page 23, Table 1***

Interventions	11a	Interventions for each group with sufficient detail to allow replication, including how and when they will be administered: ***Page 9, line 15 to Page 11, line 8***
	11b	Criteria for discontinuing or modifying allocated interventions for a given trial participant (eg, drug dose change in response to harms, participant request, or improving/worsening disease): ***Page 9, line 13*** and ***page 11 lines 17-18.***
	11c	Strategies to improve adherence to intervention protocols, and any procedures for monitoring adherence (e.g., drug tablet return, laboratory tests): ***Page 11 lines 3-8***
	11d	Relevant concomitant care and interventions that are permitted or prohibited during the trial: ***Page 9, line 18 to page 10, line 5***
Outcomes	12	Primary, secondary, and other outcomes, including the specific measurement variable (eg, systolic blood pressure), analysis metric (eg, change from baseline, final value, time to event), method of aggregation (eg, median, proportion), and time point for each outcome. Explanation of the clinical relevance of chosen efficacy and harm outcomes is strongly recommended: ***Page 11, line 21 to page 13, line 19*** and ***Page 25, Table 4***
Participant timeline	13	Time schedule of enrolment, interventions (including any run-ins and washouts), assessments, and visits for participants. A schematic diagram is highly recommended (see Figure): ***Page 24, Table 3*** and ***Page 27, Figure 1***
Sample size	14	Estimated number of participants needed to achieve study objectives and how it was determined, including clinical and statistical assumptions supporting any sample size calculations: ***Page 13, lines 21-24***
Recruitment	15	Strategies for achieving adequate participant enrolment to reach target sample size: ***Page 9, lines 1-5*** and ***Page 11, lines 3-8***

Methods: Assignment of interventions (for controlled trials)

Allocation:

Sequence generation	16a	Method of generating the allocation sequence (eg, computer-generated random numbers), and list of any factors for stratification. To reduce predictability of a random sequence, details of any planned restriction (eg, blocking) should be provided in a separate document that is unavailable to those who enrol participants or assign interventions: ***Page 14, lines 3-7***
Allocation concealment mechanism	16b	Mechanism of implementing the allocation sequence (eg, central telephone; sequentially numbered, opaque, sealed envelopes), describing any steps to conceal the sequence until interventions are assigned: ***Page 13, lines 14 to 22***

Implementation	16c	Who will generate the allocation sequence, who will enrol participants, and who will assign participants to interventions: ***Page 13, lines 14 to 22***
Blinding (masking)	17a	Who will be blinded after assignment to interventions (eg, trial participants, care providers, outcome assessors, data analysts), and how: ***Page 8, lines 7-10***
	17b	If blinded, circumstances under which unblinding is permissible, and procedure for revealing a participant's allocated intervention during the trial: ***The design is open label with*** (except for some details of the randomization procedure) ***virtually all elements of the study being unblinded. Therefore, unblinding will not occur.***

Methods: Data collection, management, and analysis

Data collection methods	18a	Plans for assessment and collection of outcome, baseline, and other trial data, including any related processes to promote data quality (eg, duplicate measurements, training of assessors) and a description of study instruments (eg, questionnaires, laboratory tests) along with their reliability and validity, if known. Reference to where data collection forms can be found, if not in the protocol: ***Page 10, line 6 to Page 11, line 8; Page 14, line 14 to Page 15, line 2;*** and ***Supplementary material 4 -- DARK-Sepsis DCF (v3.1 09Jul2023)***
	18b	Plans to promote participant retention and complete follow-up, including list of any outcome data to be collected for participants who discontinue or deviate from intervention protocols: ***Page 11, lines 3-8***
Data management	19	Plans for data entry, coding, security, and storage, including any related processes to promote data quality (eg, double data entry; range checks for data values). Reference to where details of data management procedures can be found, if not in the protocol: ***Page 14, line 14 to Page 15, line 2***
Statistical methods	20a	Statistical methods for analysing primary and secondary outcomes. Reference to where other details of the statistical analysis plan can be found, if not in the protocol: ***Page 11, line 20 to Page 13, line 19***
	20b	Methods for any additional analyses (eg, subgroup and adjusted analyses): ***Page 12, line 17 to Page 13, line 19.***
	20c	Definition of analysis population relating to protocol non-adherence (eg, as randomised analysis), and any statistical methods to handle missing data (eg, multiple imputation): ***Page 11, line 22***

Methods: Monitoring

Data monitoring	21a	Composition of data monitoring committee (DMC); summary of its role and reporting structure; statement of whether it is independent from the sponsor and competing interests; and reference to where further details about its charter can be found, if not in the protocol. Alternatively, an explanation of why a DMC is not needed: ***This trial is comparing the use of FDA-approved medications for their approved purposes and therefore will be low risk (beyond the risk inherent in treating septic shock with vasopressor agents). Based on FDA regulations, the only aspect of this trial which precludes qualification as a "minimal risk" study is the number of blood samples (>2 per week) being obtained. As such, the trial will not have a formal DMC. Instead, the two co-PIs will oversee data safety monitoring activities for the study, which will include daily patient evaluation for adverse events during the treatment phase of the study. Also see Page 15, lines 10-15.***
	21b	Description of any interim analyses and stopping guidelines, including who will have access to these interim results and make the final decision to terminate the trial: ***Page 15, lines 10-15.***
Harms	22	Plans for collecting, assessing, reporting, and managing solicited and spontaneously reported adverse events and other unintended effects of trial interventions or trial conduct: ***Page 11, lines 10-18*** and ***Page 25, Table 4***
Auditing	23	Frequency and procedures for auditing trial conduct, if any, and whether the process will be independent from investigators and the sponsor: ***No trial-specific auditing plan was created for this single-centre study, but per local ethics board regulations the trial is subject to yearly Continuing Reviews and unannounced IRB audits by the independent UNM Health Sciences Center Human Research Protections Office. Also see Page 15, lines 10-15.***
**Ethics and dissemination**		
Research ethics approval	24	Plans for seeking research ethics committee/institutional review board (REC/IRB) approval: ***Page 16, lines 5-11***
Protocol amendments	25	Plans for communicating important protocol modifications (eg, changes to eligibility criteria, outcomes, analyses) to relevant parties (e.g., investigators, REC/IRBs, trial participants, trial registries, journals, regulators): ***Page 16, lines 9-11***
Consent or assent	26a	Who will obtain informed consent or assent from potential trial participants or authorised surrogates, and how (see Item 32): ***Page 9, lines 1-9*** and ***Supplementary material 2 -- DARK-Sepsis Consent.***

	26b	Additional consent provisions for collection and use of participant data and biological specimens in ancillary studies, if applicable: ***No biological specimens will be stored beyond the period required to carry out the testing planned for this study, after which all samples will be discarded. The consent does not provide the investigators permission to carry out ancillary studies. Though no such studies are currently planned, if performed they will require separate regulatory approval and must be carried out after the data is fully de-identified in order to qualify for waiver of consent.***
Confidentiality	27	How personal information about potential and enrolled participants will be collected, shared, and maintained in order to protect confidentiality before, during, and after the trial: ***Page 15, lines 3-9***
Declaration of interests	28	Financial and other competing interests for principal investigators for the overall trial and each study site: ***Page 22, line 5***
Access to data	29	Statement of who will have access to the final trial dataset, and disclosure of contractual agreements that limit such access for investigators: ***Page 15, lines 21-22***; ***Page 16, lines 18-20*** and ***Page 22, lines 1-3***
Ancillary and post-trial care	30	Provisions, if any, for ancillary and post-trial care, and for compensation to those who suffer harm from trial participation: ***Supplementary material 1 -- DARK-Sepsis Consent, section titled, "WHAT HAPPENS IF YOU GET HURT OR SICK DURING THE STUDY?"***
Dissemination policy	31a	Plans for investigators and sponsor to communicate trial results to participants, healthcare professionals, the public, and other relevant groups (eg, via publication, reporting in results databases, or other data sharing arrangements), including any publication restrictions: ***Page 16, lines 13-16***
	31b	Authorship eligibility guidelines and any intended use of professional writers: ***Page 22, lines 12-19.***
	31c	Plans, if any, for granting public access to the full protocol, participant-level dataset, and statistical code: ***Page 16, line 18-20.***
**Appendices**		
Informed consent materials	32	Model consent form and other related documentation given to participants and authorised surrogates: ***Supplementary material 2 -- DARK-Sepsis Consent (v2 03Sept2022)***
Biological specimens	33	Plans for collection, laboratory evaluation, and storage of biological specimens for genetic or molecular analysis in the current trial and for future use in ancillary studies, if applicable: ***Page 10, line 6 to Page 11, line 2***

*It is strongly recommended that this checklist be read in conjunction with the SPIRIT 2013 Explanation & Elaboration for important clarification on the items. Amendments to the protocol should be tracked and dated. The SPIRIT checklist is copyrighted by the SPIRIT Group under the Creative Commons "Attribution-NonCommercial-NoDerivs 3.0 Unported" license.

WHO Trial Registration Data Set

1. Primary Registry and Trial Identifying Number: NCT05824767

2. Date of Registration in Primary Registry: April 24, 2023

3. Secondary Identifying Numbers: UNM Health Sciences Center Human Research Protections Office protocol #22-111

4. Source(s) of Monetary or Material Support: La Jolla Pharmaceutical Company

5. Primary Sponsor: University of New Mexico

6. Secondary Sponsor(s): N/A

7. Contact for Public Queries:
   J. Pedro Teixeira, MD
   Department of Internal Medicine
   University of New Mexico School of Medicine
   MSC10-5550
   1 University of New Mexico
   Albuquerque, New Mexico 87131
   505-272-0407
   jteixeira@salud.unm.edu

8. Contact for Scientific Queries:
   J. Pedro Teixeira, MD
   Department of Internal Medicine
   University of New Mexico School of Medicine
   MSC10-5550
   1 University of New Mexico
   Albuquerque, New Mexico 87131
   505-272-0407
   jteixeira@salud.unm.edu

9. Public Title: Serum Biomarkers to Predict Response to Angiotensin II in Septic Shock (DARK-Sepsis)

10. Scientific Title: DPP3, Angiotensin II, and Renin Kinetics in Sepsis (DARK-Sepsis) trial: serum biomarkers to predict response to angiotensin II versus standard-of-care vasopressor therapy in the treatment of septic shock, a randomized controlled trial

11. Countries of Recruitment: United States

12. Health Condition(s) or Problem(s) Studied: septic shock

13. Intervention(s):

- Randomization to angiotensin II or standard-of-care vasopressors
- Serial biomarker (renin and DPP3) levels
- See protocol paper *(Page 9, line 4 to Page 10, line 8) for additional details*

14. Key Inclusion and Exclusion Criteria:
    - See protocol paper, Table 1 for details

15. Study Type: single-center unblinded randomized controlled trial

16. Date of First Enrollment: May 11, 2023

17. Sample Size: target sample size 40 (20 patients in each arm)

18. Recruitment status of this trial: Recruiting (3 patients recruited as of July 11, 2023)

19. Primary Outcome(s): ability of baseline renin and DPP3 levels to predict vasopressor response adjusted for treatment arm (AT2 versus control) and Sequential Organ Failure Assessment (SOFA) scores.

20. Key Secondary Outcomes: see *Supplementary material 3 -- DARK-Sepsis secondary outcomes*

21. Ethics Review:
    - Approved (protocol #22-111)
    - Initial approval April 27, 2022; most recent version 2.2 approved April 27, 2023
    - University of New Mexico Health Sciences Center Human Research Protections Office, 505-272-1129, hsc-hrpo@salud.unm.edu

22. Completion date: October 30, 2024 (anticipated)

23. Summary Results: pending

24. IPD sharing statement: The final de-identified dataset will be made fully accessible upon reasonable request once the results are published and pending approval of UNM and La Jolla Pharmaceutical Company
