# Supplementary material for: The scientific rationale and study protocol for the DPP3, Angiotensin II, and Renin Kinetics in Sepsis (DARK-Sepsis) randomized controlled trial: serum biomarkers to predict response to angiotensin II versus standard-of-care vasopressor therapy in the treatment of septic shock
Source: Trials. 2024 Mar 12;25:182. doi: 10.1186/s13063-024-07995-0 (PMC10935947; doi:10.1186/s13063-024-07995-0)
Supplement: Supplementary file 2 — Additional file 2. Informed consent form approved by UNM institutional review board. [file 13063_2024_7995_MOESM2_ESM.pdf]

The University of New Mexico Health Sciences Center

Consent and Authorization to Participate in a Research Study

Key Information for "DPP3, Angiotensin II, and Renin Kinetics in Sepsis (DARK-Sepsis) pilot: serum biomarkers to predict response to angiotensin II versus standard-of-care vasopressor therapy in the treatment of septic shock, a randomized controlled pilot trial" (HRRC ID 22-111)

You are being invited to take part in a research study about the use of medications to treat septic shock.

WHAT IS THE PURPOSE, PROCEDURES, AND DURATION OF THE STUDY?

By doing this study, we hope to learn more about how to best treat septic shock (very low blood pressure caused by a serious infection). Your participation in this research will last for the duration of your time in the hospital. The purpose of this investigation is to study whether two blood tests – renin and dipeptidyl peptidase 3 (DPP3) – can be used to guide how to treat the low blood pressure in septic shock and, in particular, if these blood tests can be helpful to decide if someone should be treated with a drug called angiotensin II. Angiotensin II appears to be an effective option to raise blood pressure in septic shock, but whether renin or DPP3 levels are useful to predict that angiotensin II is more effective than other medications is not clear. This study hopes to answer that question.

WHAT ARE THE KEY REASONS YOU MIGHT CHOOSE TO VOLUNTEER FOR THIS STUDY?

Your participation will help us to learn how to best use this newer drug, angiotensin II, to treat septic shock and specifically how renin or DPP3 may be helpful in patients with septic shock treated with angiotensin II or other medications. This information will ultimately help us improve care of patients with septic shock. Currently, at the UNM Hospital, only patients who participate in this trial will have access to angiotensin II. For a complete description of benefits, refer to the Detailed Consent.

WHAT ARE THE KEY REASONS YOU MIGHT NOT CHOOSE TO VOLUNTEER FOR THIS STUDY?

You may choose not to participate because there is a slight risk of breach of confidentiality (release of my personal information), because having blood drawn can cause discomfort, because answering questions may cause stress or emotional discomfort, or because you are participating in another trial. If you chose not to participate in this trial, we will treat your septic shock with other available drugs (e.g., norepinephrine, epinephrine, vasopressin, phenylephrine, or dopamine). For a complete description of the risks and of the alternate treatment/procedures, refer to the Detailed Consent.

DO YOU HAVE TO TAKE PART IN THE STUDY?

If you decide to take part in the study, it should be because you really want to volunteer. You will not lose any services, benefits, or rights you would normally have if you choose not to volunteer.

WHAT IF YOU HAVE QUESTIONS, SUGGESTIONS OR CONCERNS?

The persons in charge of this study are Dr. J. Pedro Teixeira and Dr. Nathan Nielsen of the University of New Mexico Health Sciences Center, Department of Internal Medicine. If you have questions, suggestions, or concerns regarding this study or you want to withdraw from the study, please contact Dr. Teixeira; his contact information is 505-272-0407 or jteixeira@salud.unm.edu.

If you have any questions or concerns about your rights as a volunteer in this research, contact staff in the University of New Mexico Health Sciences Center (UNMHSC) Human Research Review Committee (HRRC) between the business hours of 8AM and 5PM, Mountain Standard Time (MST), Monday-Friday at 505-272-1129.

DETAILED CONSENT

Version 2 03Sept2022

ARE THERE REASONS WHY YOU WOULD NOT QUALIFY FOR THIS STUDY?

Individuals under the age of 18 and individuals who are not able to consent on their own and do not have an authorized person qualified to consent for them do not qualify for this study. Other medical conditions that would potentially exclude you include severe asthma or COPD; severe heart disease (new heart attack or shock from heart failure); severe disease of the aorta (aneurysm or dissection); new stroke; impairment of blood flow to the gut; active bleeding; untreated clots in leg veins or in the lungs (deep vein thrombosis, DVT, or pulmonary embolism, PE); severe liver disease; certain rare diseases (i.e., scleroderma or systemic sclerosis); certain conditions that result in compromised immune systems; allergies to angiotensin II or mannitol; pregnancy; or participation in another clinical trial.

WHERE IS THE STUDY GOING TO TAKE PLACE AND HOW LONG WILL IT LAST?

The study will be conducted while you are hospitalized at University of New Mexico Hospital (UNMH). The research portion of this study will be conducted at UNM Health Science Center (UNM HSC). Your participation in the study will last the duration of your hospital admission.

The entire study will take place over 1 year. We anticipate including a total of 40 patients in the trial (20 in the treatment arm and 20 in the control arm).

WHAT WILL YOU BE ASKED TO DO?

By giving your consent, you allow us to do 3 things: (1) randomly assign you to one of two vasopressor treatments for septic shock, (2) collect your information, and (3) collect your blood for research.

The purpose of this investigation is to study whether two blood tests (renin and DPP3) can be used to guide how to treat the low blood pressure in septic shock. In particular, prior studies suggest that high blood levels of renin or DPP3 may be helpful to predict survival in septic shock and may specifically be useful in determining if the medication angiotensin II will be very helpful to increase blood pressure in septic shock. Angiotensin II is the synthetic form of a natural substance (a hormone) found in the body that increases blood pressure. Angiotensin II was approved to treat septic shock by the FDA in 2017. Angiotensin II appears to be an effective option to raise blood pressure in septic shock, but whether blood renin or DPP3 levels are useful to predict that angiotensin II is more effective than other medications is not clear. Because renin and DPP3 may be helpful to predict outcomes in patients with septic shock treated with medications other than angiotensin II, our study will have a control group in which renin and DPP3 levels are measured but angiotensin II is not used.

Vasopressors are medications used to increase blood pressure in patients with shock (severely low blood pressure). As part of standard of care, all patients in this trial will be treated first with norepinephrine for septic shock. Norepinephrine is the most commonly used vasopressor medication to treat septic shock and is considered the best first-line therapy for septic shock. Also, as part of standard of care, patients in this study will have a central venous catheter (a “central line”) and an arterial catheter (an “arterial line”) in place. A catheter is a small plastic tube inserted into a large vein or artery, similar to a regular “IV”, that allows us to give medications safely or to monitor your blood pressure in a continuous way. Central venous catheters are considered the safest way to administer vasopressors, particularly at higher doses. Similarly, arterial catheters allow for very precise (second-to-second) monitoring of blood pressure and are consider standard of care for moderate-to-severe septic shock. Patients will be included in this study if their blood pressure remains low despite norepinephrine. Angiotensin II,

the drug being studied, has been shown to be safe and effective at raising blood pressure when norepinephrine alone is not enough, but whether blood renin or DPP3 levels can reliably predict that angiotensin II will be effective is not clear. By giving us your consent, you allow us to randomly select either angiotensin II in addition to norepinephrine (in the treatment group) or other available vasopressors (in the control group) to treat septic shock.

By giving your consent, you also agree to allow us to collect information from you during the hospitalization and review or obtain medical records from this hospitalization so we can determine the duration, outcome, and severity of your illness; any underlying illness you have; and medications you have received.

Finally, by giving your consent, you allow us to draw blood to better study the effect of angiotensin II compared to standard of care. Specifically, preliminary studies suggest that two blood tests (renin and DPP3 levels) may help determine who benefits from treatment with angiotensin II. In this study, we are going to test if this is true by measuring renin and DPP3 levels in the blood of patients treated with angiotensin II. Other studies suggest that renin or DPP3 levels may be used do to predict survival in all patients with septic shock, including those not treated with angiotensin II. In our control group, we will measure renin and DPP3 levels in patients that are not treated with angiotensin II, which will help us tell if measuring renin and DPP3 levels gives us specific information about the usefulness of angiotensin II or if renin and DPP3 levels instead tell us if someone is likely to survive septic shock regardless of treatment. Because some studies suggest that changes in blood renin and DPP3 levels are more important than testing just one renin or DPP3 level, we will obtain renin and DPP3 levels multiple times in both arms of the study.

To measure these renin and DPP3 levels, we will need to collect an extra 3 milliliters (less than one teaspoon) of blood with each blood draw and will obtain blood up to 6 times over the first 4 days of the study. If you are assigned to the angiotensin II (treatment) arm, you will have up to 6 blood samples obtained; if you are assigned to the control (standard of care arm), you will have up to 5 blood samples obtained. Since we will only be enrolling patients in this study who have central venous catheters and arterial catheters in place, we can use these catheters to collect the blood. Collecting blood from catheters is a painless procedure but, in the rare instance that both of these catheters unexpectedly malfunction, we might have to collect blood by phlebotomy (placing a needle into a vein).

WHAT ARE THE POSSIBLE RISKS AND DISCOMFORTS?

You will be included in the study only if your medical providers caring for you in the intensive care unit believe that you are a good candidate to be treated with angiotensin II. Angiotensin II is an FDA-approved treatment for septic shock. This approval is based on a trial which concluded that angiotensin II is likely to be a safe and effective treatment for septic shock.

However, the disease septic shock itself carries a significant risk itself of adverse events. In addition, though necessary to treat septic shock, all vasopressor medications carry risk of adverse events. Based on prior studies, these adverse events may include but are not limited to:

- New clot in artery or vein (arterial thrombosis or venous thromboembolism)
- New fast or abnormal heart rhythm (tachycardia or atrial fibrillation)
- Build-up of acid in the blood (lactic acidosis)
- Poor blood flow and injury to multiple organs, including:
    - The tips of your fingers or toes (peripheral limb or digit ischemia)
    - Gut (intestinal ischemia)
    - Heart (heart attack)
    - Brain (stroke)

    - Kidneys (kidney injury and failure)
- Low levels of blood platelets (thrombocytopenia)
- High blood sugar (hyperglycemia)
- New infection

These adverse events can occur with any patient with septic shock being treated with any vasopressor treatment, including patients who do not choose to participate in the study who are treated with the vasopressor treatments available outside of this study (such as norepinephrine, epinephrine, vasopressin, phenylephrine, or dopamine). Right now, it is not clear that any of these different treatments increase the risk of these adverse events more than the others.

Regardless of whether you choose to participate in this trial, your care in the ICU at UNM Hospital will be provided according to best practice guidelines to minimize these risks and to maximize the chance of a good outcome, but you are encouraged to ask your medical providers or the investigators about these risks of septic shock and its treatment.

If you are enrolled in this study, there is a slight possibility that you may experience a loss of confidentiality regarding your medical records. In order to protect the privacy of your clinical information, the research team will take measures like restricting access to your records and using security passwords and locked cabinets for storing the data. All data collected from this study will be retained and stored for a maximum of seven years after closure of the study.

As mentioned above, we will collect blood for this study. The amount of blood collected is small (a half-teaspoon or less at a time) and equal to or less than the amount blood that is normally taken daily from patients while hospitalized. Because we will require patients in this study to have a central venous catheter and an arterial catheter in place, we will plan to use these catheters to painlessly obtain the blood. However, in the rare instance that both catheters malfunction, we will obtain by blood by phlebotomy (needle placed into a vein). The drawing of blood with a needle from an arm vein usually causes mild pain (90%) and rarely causes a bruise (1%). There is a remote chance of fainting (less than 0.1%) and a very remote chance of infection (less than 0.01%).

There is always a chance that any medical treatment can harm you. The research treatments in this study are no different. In addition to risks described in this consent, you may experience a previously unknown risk or side effect.

If you choose not to participate in this trial, your condition will be treated with other medications (other vasopressors) available outside this study (potentially including norepinephrine, vasopressin, epinephrine, phenylephrine, or dopamine).

WILL YOU BENEFIT FROM TAKING PART IN THIS STUDY?

We do not know if you will get any benefit from participating in this study. As above, angiotensin II appears to be effective when used to increase blood pressure in septic shock. Though we need more data to be certain, earlier studies have suggested that angiotensin II might be especially effective for certain types of patients with septic shock (e.g., patients with high blood renin or DPP3 levels or patients with injured lungs or injured kidneys). Regardless, if you take part in this study, information learned may help others with your condition.

WHAT WILL IT COST YOU TO PARTICIPATE?

There are no additional costs associated with taking part in the study.

WHO WILL SEE THE INFORMATION THAT YOU GIVE?

When we write about or share the results from the study, we will write about the combined information. We will keep your name and other identifying information private. We will make every effort to prevent anyone who is not on the research team from knowing that you gave information or what the information is.

All information about your taking part in this study will be kept confidential. If you decide to participate, your records will be managed by the research team. If you participate, your medical records may be inspected by study personnel and the ethics committee at UNM. Any identifiable information, such as your name, will not be recorded on the study forms and will be replaced by a unique coded identifier to provide additional added confidentiality.

We will use a tool called REDCap to store data for this study. REDCap is a secure, web-based program to capture and store data at the University of New Mexico. Please be aware, while we make every effort to safeguard your data once received on servers via REDCap, given the nature of online servers, as with anything involving the internet, we can never fully guarantee the confidentiality of the data while still in route to the server.

A description of this clinical trial will be available on ClinicalTrials.gov as required by U.S. Law. This website will not include information that can identify you. At most, the website will include a summary of the results. You can search this website at any time.

CAN YOU CHOOSE TO WITHDRAW FROM THE STUDY EARLY?

You can choose to leave the study at any time. You will not be treated differently if you decide to stop taking part in the study.

If you choose to leave the study early, data collected until that point will remain in the study database and may not be removed, but no new data will be collected.

The investigators conducting the study may need to remove you from the study. The study medication will no longer be provided to you and will not be available for purchase. Though this is not anticipated, this could occur for a number of reasons. For example, we may need to remove you from the study if you develop or we discover a condition that would disqualify you from the study. If at any point the investigators believe that your participation in the study has more risk than benefit to you, you will be removed from the study.

Because all patients in this study will be in an intensive care unit at UNM Hospital and will already be on vasopressor treatment before starting the trial, all the patients will already be under close monitoring (with frequent or continuous measurements of vital signs such as blood pressure and heart rate, frequent assessment of symptoms by nurses, frequent adjustments of medications, and frequent laboratory testing). Because of this, no additional specific monitoring will be needed for this study on top of the close monitoring being provided as part of the standard of care in the intensive care unit, which will allow us to safely adjust the dose of the medications being used, angiotensin II in the treatment arm or other vasopressor(s) in the control arm. Though we do not expect this, we will also use all this information provided by this high level of monitoring to help us decide if we need to remove you from the study for safety reasons.

ARE YOU PARTICIPATING, OR CAN YOU PARTICIPATE, IN ANOTHER RESEACH STUDY AT THE SAME TIME AS PARTICIPATING IN THIS ONE?

You may not take part in this study if you are currently involved in another research study that is evaluating another treatment or intervention, but participating in a separate non-interventional (i.e., observational) trial is allowed. It is important to let the investigator and your doctor know if you are in another research study.

WHAT HAPPENS IF YOU GET HURT OR SICK DURING THE STUDY?

If you believe you are hurt or you get sick because of something that is due to this study, you should call Dr. Teixeira at 505-272-0407 immediately.

It is important for you to understand that the UNM does not have funds set aside to pay for the cost of any care or treatment that might be necessary because you get hurt or sick while taking part in this study. Also, the UNM will not pay for any wages you may lose if you are harmed by this study.

Medical costs related to your care and treatment because of study-related harm will not be reimbursed by this study.

WILL I BE PAID FOR PARTICIPATING IN THIS STUDY?

You will not receive any financial rewards or payment for taking part in the study. Because this study is analyzing a treatment (angiotensin II) that is FDA-approved for the condition being studied (septic shock) and the ideas being tested (e.g., the idea that renin or DPP3 levels may be a good way to figure out if angiotensin II will be a helpful treatment) are based on prior scientifically sound research, the risk of participating in this study is reasonable despite the lack of payment.

WHAT IF NEW INFORMATION IS LEARNED DURING THE STUDY THAT MIGHT AFFECT YOUR DECISION TO PARTICIPATE?

You will be informed if the investigators learn new information that could change your mind about staying in the study. You may be asked to sign a new informed consent form if the information is provided to you after you have joined the study.

WILL YOU BE GIVEN INDIVIDUAL RESULTS FROM THE RESARCH TESTS?

In this study, the bulk of the data collected will be the results of tests that are being done already for the purposes of your medical care. The blood collected will be used to measure the levels of substances called renin and DPP3, but these results will not be shared with you or your medical team. We are measuring renin and DPP3 levels because there is a theory that doing so can be helpful in treating septic shock, but this theory has not been proven and, for this reason, sharing the information with you or your medical team currently has no benefit.

FUTURE USE OF YOUR PROTECTED HEALTH INFORMATION

Your information or samples collected for this study will NOT be used or shared for future research studies, even if we remove the identifiable information like your name, medical record number, or date of birth.

HIPAA AUTHORIZATION FOR USE AND DISCLOSURE OF YOUR PROTECTED HEALTH INFORMATIN (PHI).

As part of this study, we will be collecting health information about you and sharing it with others without identifying you. Links to your name and identifiable health information will be kept on a secure online database (REDCap) and will not be shared with others. This information is "protected" because it is identifiable or "linked" to you. Any information which does identify you is kept confidential and complies with the Health Insurance Portability and Accountability Act (HIPAA).

PROTECTED HEALTH INFORMATION (PHI)

By signing this Consent Document, as described in this consent form, you are allowing the investigators and other authorized personnel to use your protected health information for the purposes of this study. This information includes results of physical exams, medical history, and results of blood tests.

In addition to researchers and staff at UNM HSC and other groups listed in this form, there is a chance that your health information may be shared (re-disclosed) outside of the research study and no longer be protected by federal privacy laws. Examples of this include health oversight activities and public health measures, safety, monitors, other sites in the study, companies that sponsor this study, and government agencies such as Food and Drug Administration (FDA).

Right to Withdraw Your Authorization

Your authorization for the use and disclosure of your health information for this study shall not expire unless you cancel this authorization. This is because the information used and created during the study may be analyzed for many years and it is not possible to know when this will be complete. Your health information will be used or disclosed as long as it is needed for this study. However, you may withdraw your authorization at any time provided you notify the UNM investigators in writing. To do this, please send letter notifying them of your withdrawal to:

Dr. J. Pedro Teixeira
MSC10 5550
1 University of New Mexico
Albuquerque New Mexico 87131

While you are in the hospital, you may also withdraw by speaking with a member of the research team or by calling Dr. Teixeira at 505-272-0407. You may also be withdrawn from the study if the investigator feels it is in your best interest to withdraw you from the study.

Please be aware that the research team will not be required to destroy or retrieve any of your health information that has already been used or shared before the date that your withdrawal is received.

The researchers agree to only share your health information with the people listed in this document. Should your health information be released to anyone that is not regulated by the privacy law, your health information may be shared with others without your permission; however, the use of your health information would still be regulated by applicable federal and state laws.

You may not be allowed to participate in the research study if you do not sign this form. If you decide not to sign this form, it will not affect your:

- Current or future healthcare at the University of New Mexico;
- Current or future payments to the University of New Mexico;
- Ability to enroll in any health plans (if applicable); or
- Eligibility for benefits (if applicable).

After signing the form, you can change your mind and NOT let the researcher(s) collect or release your health information (revoke the Authorization). If you revoke the authorization:

- You will send a written letter to Dr. J. Pedro Teixeira, MSC10 5550, 1 University of New Mexico, Albuquerque, NM 87131-0001 to inform him of your decision.
- Researchers may use and release your health information already collected for this research study.
- Your protected health information may still be used and released should you have a bad reaction (adverse event).

The use and sharing of your information have no time limit.

If you have not already received a copy of the Privacy Notice, you may request one. If you have any questions about your privacy rights, you should contact the University of New Mexico Health Sciences Privacy Officer between the business hours of 8am and 5pm Mountain Pacific Time, Monday-Friday at (505) 272-1493.

WHAT IF YOU HAVE QUESTIONS LATER ON?

If you have questions related to this research, you can contact Dr. Teixeira at 505-272-0407. If you have questions, problems, or concerns about your participation in the research and would like to speak with someone other than the research team, you may call the UNM HSC Human Research Protections Office at (505) 272 1129. You may also call this number if you have questions about your rights as a research subject. For more information, you may also access the HRRC website at http://hsc.unm.edu/som/research/hrrc/ or http://hsc.unm.edu/research/hrpo/.

INFORMED CONSENT SIGNATURE PAGE (HRRC ID 22-111)

You are participating or are authorized to act on behalf of the participant. This consent includes the following:

- Key Information Page
- Detailed Consent

You will receive a copy of this consent form after it has been signed.

____________________________________ ________________________

Signature of research subject **Date**

**research subject's legal representative may be allowed to provide consent by phone*

____

Printed name of research subject

____

**If applicable, printed name of research subject's legal representative*

____________________________________ ________________________

Signature of legal representative, *if applicable* **Date**

*If applicable, please explain Representative's relationship to subject and include a description of representative's authority to act on behalf of subject:

____________________________________ ________________________

Printed name of [authorized] person obtaining informed consent/HIPAA Authorization Date

____

Signature of [authorized] person obtaining informed consent/HIPAA Authorization
