# Supplementary material for: The scientific rationale and study protocol for the DPP3, Angiotensin II, and Renin Kinetics in Sepsis (DARK-Sepsis) randomized controlled trial: serum biomarkers to predict response to angiotensin II versus standard-of-care vasopressor therapy in the treatment of septic shock
Source: Trials. 2024 Mar 12;25:182. doi: 10.1186/s13063-024-07995-0 (PMC10935947; doi:10.1186/s13063-024-07995-0)
Supplement: Supplementary file 3 — Additional file 3. University of New Mexico Hospital Nursing Department Titration Guideline. [file 13063_2024_7995_MOESM3_ESM.pdf]

HOSPITALS

Applies To: **UNM Hospitals**<br>Responsible Department: Nursing<br>Effective Date: 03/11/2020

Title: **Titration of Medications**					**Guideline**
**Patient Age Group:**	( ) N/A	(X) All Ages	( ) Newborns	( ) Pediatric	( ) Adult

DESCRIPTION/OVERVIEW

The University of New Mexico Hospitals provides standards for the titration of medications to provide safe and effective treatment for patients. This guideline does not include medication titration during emergent situations; in emergent patient care situations there should not be a delay in instituting the appropriate emergency treatment.

REFERENCES

Jentzer, J. C., Coons, J. C., Link, C. B., & Schmidhofer, M. (2015). Pharmacotherapy update on the use of vasopressors and inotropes in the intensive care unit. *Journal of Cardiovascular Pharmacology and Therapeutics, 20*(3), 249–260. https://doi.org/10.1177/1074248414559838

Lexicomp Online. Hudson, Ohio: Lexi-Comp, Inc; Accessed 12/25/2019.

Lexicomp Online. Pediatric and neonatal Lexi-Drugs. Hudson, Ohio: Lexi-Comp, Inc. Retrieved 12/25/2019 from http://online.lexi.com.

Micromedex. Ann Arbor (MI): Truven Health Analytics. Retrieved 12/25/2019 from 12/25/2019.

National High Blood Pressure Education Program. (2004). The seventh report of the Joint National Committee on prevention, detection, evaluation, and treatment of high blood pressure. Retrieved from http://www.ncbi.nlm.nih.gov/books/NBK9630/

Phelps, S. J., Hagemann, T. M., Lee, K. R., & Thompson, A. J. (2018). Pediatric injectable drugs: The teddy bear book (11thed.). American Society of Health System.

Rhoney, D., & Peacock, W. F. (2009a). Intravenous therapy for hypertensive emergencies, part 1. American Journal of Health-System Pharmacy: *AJHP: Official Journal of the American Society of Health-System Pharmacists, 66*(15), 1343–1352. https://doi.org/10.2146/ajhp080348.p1

Rhoney, D., & Peacock, W. F. (2009b). Intravenous therapy for hypertensive emergencies, part 2. American Journal of Health-System Pharmacy: *AJHP: Official Journal of the American Society of Health-System Pharmacists, 66*(16), 1448–1457. https://doi.org/10.2146/ajhp080348.p2

Russell, J. A. (2019). Vasopressor therapy in critically ill patients with shock. *Intensive Care Medicine, 45*(11), 1503–1517. https://doi.org/10.1007/s00134-019-05801-z

The Joint Commission standards manual medication management chapter: MM.04.01.01

Whelton, P. K., Carey, R. M., Aronow, W. S., Casey, D. E., Collins, K. J., Dennison Himmelfarb, C., … Wright, J. T. (2018). 2017 ACC/AHA/AAPA/ABC/ACPM/AGS/APhA/ASH/ASPC/NMA/ PCNA guideline for the prevention, detection, evaluation, and management of high blood pressure in adults: Executive summary: A report of the American College of Cardiology/American Heart Association Task Force on Clinical Practice Guidelines. *Hypertension 71*(6), 1269–1324. https://doi.org/10.1161/HYP.0000000000000066

AREAS OF RESPONSIBILITY

This guideline applies to all Licensed Independent Practitioners (LIPs) who order or administer medications within their scope of practice, as determined by licensure and education.

PROCEDURE

In emergent situations patient care is the priority. The LIP may issue a verbal order for medications in the case of an emergency situation prior to documenting in the electronic medical record (EMR).

1. **Orders for titratable medications may be ordered by:**
   - 1.1. The LIP placing an order for the medication which includes the initial dose and each dose (rate) change thereafter.
   - 1.2. The LIP placing an order for the medication which includes the initial dose, and placing the appropriate "Medication Titration Parameter" order. The nurse will titrate the medication based on the attached titration guidelines.
   - 1.3. The LIP placing an order for the medication to be titrated and detailing the titration details on the order in the comment field.
   - 1.4. The required elements for a titration order include:
     - 1.4.1. Medication name
     - 1.4.2. Medication route
     - 1.4.3. Initial or starting rate of infusion (dose/min)
     - 1.4.4. Incremental units the rate can be increased or decreased
     - 1.4.5. Frequency for incremental doses (how often dose/rate can be increased or decreased)
     - 1.4.6. Maximum rate (dose) of infusion
     - 1.4.7. Objective clinical endpoint/parameter (sedation score, confusion assessment method, etc.) and holding parameters (when applicable)
   - 1.5. The last medication added is the first medication titrated off unless otherwise specified by the LIP in the medication order or the appropriate Medication Titration Parameter order.
   - 1.6. Nursing communication orders are not medication orders and may not be used to modify or change medication therapy including but not limited to medication, route, dose, rate, maximum dose, notify LIP parameters, and clinical parameters.
2. **Titration and monitoring**
   - 2.1. The nurse will titrate the medication based on the order placed by the LIP and the hospital approved guideline.
     - 2.1.1. The nurse should be able to identify the location of appropriate titration guideline in medical record.
   - 2.2. The lowest effective dose of the medication ordered, which achieves the clinical response, will be utilized.
   - 2.3. If the medication dose reaches the maximum dose, or the "call LIP" dose, the nurse will contact the LIP and notify them of the patient's status. If the LIP decides to exceed the maximum dose listed, the new maximum or "contact LIP" dose must be specified in the medication order.
     - 2.3.1. In emergent situations a verbal order may be obtained until the LIP is available to place a medication order.
   - 2.4. Vital signs are routinely monitored and documented based on the standard for the patient care area.
     - 2.4.1. Arterial line for continuous blood pressure monitoring should be considered for all patients requiring vasoactive continuous infusions.
   - 2.5. If the medication is being actively titrated, the patient's vital signs should be monitored per the titration guideline until the patient is at the desired clinical parameter for the designated time. If the patient is not in an ICU setting and is requiring frequent titration adjustments the

LIP should be notified to consider transfer to an area, such as an ICU, which allows more frequent monitoring.

2.6. All rate changes and vitals will be documented in the patient's electronic medical record.

DEFINITIONS

Titration order: Medication order in which the dose is either progressively increased or decreased in response to the patient's status.
Taper order: Medication order in which the dose is decreased by a particular amount with each dosing interval.
Dosing interval: The minimum time between dose changes or titrations
Dose increment: The dose change that occurs at each titration
Clinical parameter: Provider specified physiologic goal for titration
Medication Titration Parameter Order: The order that the provider places specifying whether a medication should be titrated and the clinical goal for titration
ACLS: Advanced Cardiac Life Support
PALS: Pediatric Advanced Cardiac Life Support
LIP: Licensed Independent Practitioner or credentialed Physician Assistant

SUMMARY OF CHANGES

11/2018: 1.4 updated to clarify titration order
1/2020: Updated titrations on Attachment D

RESOURCES/TRAINING

Resource/Dept	Contact Information
Unit UBE	As per each unit
Clinical education	As per each unit

DOCUMENT APPROVAL & TRACKING

Item	Contact	Date	Approval
**Owner**	Chair, Critical Care Committee, UNM Hospitals		
**Consultant(s)**	ED/ICU Clinical Pharmacy Lead;		
**Committee(s)**	Critical Care Committee, ED Operations Committee, Adult Nursing/Pharmacy Committee, Nurse Practice Council, UNM Hospitals PP&G Committee		Y
**Nursing Officer**	Chief Nursing Officer, UNM Hospitals		Y
**Medical Director/Officer**	Chief Medical Officer, UNM Hospitals		Y
**Human Resources**	Chief Human Resources Officer, UNM Hospitals		Y
**Finance**	Chief Financial Officer, UNM Hospitals		Y
**Official Approver**	Chief Nursing Officer, UNM Hospitals		Y
**Official Signature**	**On SharePoint**	Date: 03/11/2020	
**Effective Date**		03/11/2020	

ATTACHMENTS

Attachment A: Newborn ICU Titration Guidelines
Attachment B: Pediatric ICU and Pediatric ED Titration Guidelines

Attachment C: Adult Subacute Care Unit and Adult ED Titration Guidelines
Attachment D: Adult ICU and Adult ED Titration Guidelines

Attachment A: Newborn ICU Titration Guidelines

		Below Goal	Provider specified goal	Above Goal
**Dobutamine** (MAP)	**Initial Dose**	5mcg/kg/min	15 minutes x 3, then hourly (MAX RATE 20mcg/kg/min)	15 minutes
	**Minimum Interval**	15 minutes		
	**Titrate by**	↑ 1 mcg/kg/min		↓ 1 mcg/kg/min
**Dopamine** (MAP)	**Initial Dose**	5 mcg/kg/min	15 minutes x 3, then 15 minutes x 2, then hourly (MAX RATE 20mcg/kg/min)	15minutes
	**Minimum Interval**	15 minutes		
	**Titrate by**	↑ 1mcg/kg/min		↓ 1mcg/kg/min
**Epinephrine** (MAP)	**Initial Dose**	0.05 mcg/kg/min	15 minutes x 3 then hourly (MAX RATE 1mcg/kg/min)	15 minutes
	**Minimum Interval**	15 minutes		
	**Titrate by**	↑ 0.1mcg/kg/min		↓ 0.1mcg/kg/min

Attachment B: Pediatric ICU and Pediatric ED Titration Guidelines

VASOPRESSORS

Medications that are titrated by the provider only: vasopressin

		Below goal	Provider specified goal	Above goal
**Norepinephrine** (MAP)	**Initial Dose**	0.03 mcg/kg/min	15 minutes (MAX RATE 1 mcg/kg/min)	5 minutes
	**Min Interval**	5 minutes		
	**Titrate by**	↑ 0.01 mcg/kg/min		↓ 0.01 mcg/kg/min
**Epinephrine** (MAP)	**Initial Dose**	0.03 mcg/kg/min	15 minutes (MAX RATE 1 mcg/kg/min)	5 minutes
	**Min Interval**	5 minutes		
	**Titrate by**	↑ 0.01 mcg/kg/min		↓ 0.01mcg/kg/min
**Phenylephrine** (MAP)	**Initial Dose**	0.05 mcg/kg/min	15 minutes (MAX RATE 1 mcg/kg/min)	5 minutes
	**Min Interval**	5 minutes		
	**Titrate by**	↑ 0.01 mcg/kg/min		↓ 0.01 mcg/kg/min
**Dopamine** (MAP)	**Initial Dose**	3 mcg/kg/min	15 minutes (MAX RATE 20 mcg/kg/min)	15 minutes
	**Min Interval**	5 minutes		
	**Titrate by**	↑ 1 mcg/kg/min		↓ 1 mcg/kg/min

INOTROPES

Medications that are titrated by the provider only: milrinone

		Below Goal	Provider specified goal	Above Goal
**Dobutamine** (MAP)	**Initial Dose**	5 mcg/kg/min	5 minutes x 3, then 15 minutes x 2, then hourly (MAX RATE 20 mcg/kg/min)	15 minutes
	**Min Interval**	5 minutes		
	**Titrate by**	↑ 1 mcg/kg/min		↓ 1 mcg/kg/min

ANTIHYPERTENSIVES

Medications that are titrated by the provider only: diltiazem, fenoldopam, isoproterenol, nesiritide

		Above Goal	Provider specified goal	Below Goal
**Esmolol** (BP, HR)	**Initial Rate**	50 mcg/kg/min	5 minutes x 3, 15 minutes x 2 then hourly (MAX RATE 300 mcg/kg/min)	5 minutes
	**Min Interval**	5 minutes		↓ 25 mcg/kg/min
	**Titrate by**	↑ 25 mcg/kg/min		
**Nitroglycerin** (SBP)	**Initial Rate**	0.5 mcg/kg/min	5 minutes x 3, then 15 minutes x 2, then hourly (MAX RATE 5 mcg/kg/min)	5 minutes
	**Min Interval**	5 minutes		
	**Titrate by**	↑ 0.5 mcg/kg/min		↓ 0.5 mcg/kg/min
**Nitroprusside** (SBP)	**Initial Rate**	0.5 mcg/kg/min	5 minutes x 3, then 15 minutes x 2, then hourly (MAX RATE 5 mcg/kg/min)	5 minutes
	**Min Interval**	5 minutes		
	**Titrate by**	↑ 0.5 mcg/kg/min		↓ 0.5 mcg/kg/min
**Nicardipine** (SBP)	**Initial Rate**	0.5 mcg/kg/min	5 minutes x 3 then every 15 minutes (MAX RATE 5 mcg/kg/min)	15 minutes
	**Min Interval**	15 minutes		
	**Titrate by**	↑ 0.5 mcg/kg/min		↓ 0.5 mcg/kg/min

TITRATION BY URINE OUTPUT

Medications that are titrated by the provider only: bumetanide, furosemide, vasopressin

Attachment C: Adult Subacute Care Unit and Adult ED Titration Guidelines

Refer to the "Continuous and Intermittent IV Infusion Administration Chart" for administration restrictions by unit
Monitoring:

- All patients receiving any of the listed continuous infusions require cardiac monitoring.
- Vital signs will be assessed every 2 hours once the patient is at stated goal for vasopressors, inotropes, and antihypertensives.

VASOPRESSORS

	MAP Goal	Below Goal	Provider specified goal	Above Goal
Dopamine	Initial Dose	2.5 mcg/kg/min	Monitor 15 minutes x 3 then every 2 hours if patient at goal (MAX RATE 5 mcg/kg/min)	Provider to titrate; provider will change the dose/rate in CPOE
	Min Interval	Provider to titrate; provider will change the dose/rate in CPOE		
	Titrate by			

INOTROPES

		Below Goal	Provider specified goal	Above Goal
Dobutamine	Initial Dose	2.5 mcg/kg/min	Monitor 15 minutes x 3 then every 2 hours if patient at goal (MAX RATE 5 mcg/kg/min)	Provider to titrate; provider will change the dose/rate in CPOE
	Min Interval	Provider to titrate; provider will change the dose/rate in CPOE		
	Titrate by			
Milrinone	Initial Dose	0.2 mcg/kg/min	Monitor 15 minutes x 3 then every 2 hours if patient at goal (MAX RATE = 0.75 mcg/kg/min)	Provider to titrate; provider will change the dose/rate in CPOE
	Min Interval	Provider to titrate; provider will change the dose/rate in CPOE		
	Titrate by			

ANTIHYPERTENSIVES

In general, blood pressure should not be lowered by greater than 10-20% in the first hour and up to 35% over the first 24 hours unless otherwise specified by the provider.

		Above Goal	Provider specified goal	Below Goal
Diltiazem (BP, HR)	Initial Bolus	0.25 mg/kg (up to 20mg per provider order)	15 minutes x 3, then every 2 hours if patient at goal (MAX RATE 15 mg/hr)	N/A
	Initial Rate	2.5 mg/hr		N/A
	Min Interval	15 minutes		15 minutes
	Titrate by	↑ 2.5 mg/hr		↓ 2.5 mg/hr
Nitroglycerin (BP, Chest Pain)	Initial Rate	5 mcg/min	5 minutes x 3, then 15 minutes x 2, then every 2 hours if patient at goal (MAX RATE 200 mcg/min)	N/A
	Min Interval	BP: 15 minutes<br>Chest Pain: 3 minutes		BP: 15 minutes<br>Chest Pain: 5 minutes
	Titrate by	Dose <20 mcg/min: ↑ 5 mcg/min<br>Dose ≥20 mcg/min: ↑10 mcg/min		↓ 10 mcg/min

TITRATION BY URINE OUTPUT

		Below Goal	Provider specified goal	Above Goal	Notify Provider
Bumetanide (UOP)	Initial Rate	0.5 mg/hr	Monitor every 4 hours (MAX RATE 2 mg/hr)	N/A	▪ UOP is greater than goal<br>▪ Dose of 1.5mg/hr is reached<br>▪ SBP <90 mmHg
	Min Interval	4 hour		4 hour	
	Titrate by	↑ 0.25 mg/hr		↓ 0.25 mg/hr	
Furosemide (UOP)	Initial Rate	10 mg/hr	Monitor every 4 hours (MAX RATE 40 mg/hr)	N/A	▪ UOP is greater than goal<br>▪ Dose of 20mg/hr is reached<br>▪ SBP <90 mmHg
	Min Interval	4 hour		4 hour	
	Titrate by	↑ 5 mg/hr		↓ 5 mg/hr	

<u>**Attachment D: Adult ICU and Adult ED Titration Guidelines**</u>

VASOPRESSORS*

*****For patients > 150 kg max weight at 150 kg*****

	MAP Goal	≥20mmHg below goal	10-19 mmHg below goal	1-9 mmHg below goal	Provider specified goal	1-9 mmHg above goal	10-19 mmHg above goal	≥20 mmHg above goal
**Norepinephrine (mcg/kg/min)**	**Initial Dose**	0.3 mcg/kg/min	0.15 mcg/kg/min	0.05 mcg/kg/min	15 minutes (MAX 2 mcg/kg/min)	15 minutes	10 minutes	5 minutes
	**Min Interval**	1 minute	5 minutes	10 minutes				
	**Titrate by**	↑ 0.1 mcg/kg/min	↑ 0.05 mcg/kg/min	↑ 0.02 mcg/kg/min		1st: Reassess 2nd: ↓0.02 mcg/kg/min	↓ 0.05 mcg/kg/min	↓ 0.1 mcg/kg/min
	**Notify provider at 0.2 mcg/kg/min, Attending approval required for doses greater than 0.5 mcg/kg/min**							
**Vasopressin (units/min)**	**Initial Dose**	0.04 units/min	0.03 units/min	0.03 units/min	15 minutes (MAX 0.04 units/min)	15 minutes	10 minutes	5 minutes
	**Min Interval**	5 minute	5 minutes	10 minutes				
	**Titrate by**	Add additional vasopressor	↑ 0.01 unit/min	↑ 0.01 unit/min		↓ 0.02 unit/min	↓ 0.02 unit/min	Hold
**Epinephrine (mcg/kg/min)**	**Initial Dose**	0.3 mcg/kg/min	0.15 mcg/kg/min	0.05 mcg/kg/min	15 minutes (MAX 2 mcg/kg/min)	15 minutes	10 minutes	5 minutes
	**Min Interval**	1 minute	5 minutes	10 minutes				
	**Titrate by**	↑ 0.1 mcg/kg/min	↑ 0.05 mcg/kg/min	↑ 0.02 mcg/kg/min		1st: Reassess 2nd: ↓0.02 mcg/kg/min	↓ 0.05 mcg/kg/min	↓ 0.1 mcg/kg/min
	**Notify provider at 0.2 mcg/kg/min, Attending approval required for doses greater than 0.5 mcg/kg/min**							
**Phenylephrine (mcg/kg/min)**	**Initial Dose**	1 mcg/kg/min	0.75 mcg/kg/min	0.5 mcg/kg/min	15 minutes (MAX 5 mcg/kg/min)	15 minutes	10 minutes	5 minutes
	**Min Interval**	1 minute	5 minutes	10 minutes				
	**Titrate by**	↑ 0.5 mcg/kg/min	↑ 0.5 mcg/kg/min	↑ 0.25 mcg/kg/min		1st: Reassess 2nd: ↓0.25 mcg/kg/min	↓ 0.5 mcg/kg/min	↓ 0.5 mcg/kg/min
	**Notify provider at 1 mcg/kg/min, Attending approval required for doses greater than 2.5 mcg/kg/min**							
**Dopamine (mcg/kg/min)**	**Initial Dose**	7.5 mcg/kg/min	5 mcg/kg/min	2.5 mcg/kg/min	15 minutes (MAX 20 mcg/kg/min)	15 minutes	10 minutes	5 minutes
	**Min Interval**	5 minutes	5 minutes	10 minutes				
	**Titrate by**	↑ 5 mcg/kg/min	↑ 2.5 mcg/kg/min	↑ 2.5 mcg/kg/min		↓ 2.5 mcg/kg/min	↓ 2.5 mcg/kg/min	↓ 5 mcg/kg/min
**Angiotensin II (ng/kg/min)**	**Initial Dose**	20 ng/kg/min	10 ng/kg/min	10 ng/kg/min	15 minutes (MAX 40 ng/kg/min)	15 minutes	10 minutes	5 minutes
	**Min Interval**	5 minutes	5 minutes	5 minutes				
	**Titrate by**	↑ 10 ng/kg/min	↑ 5 ng/kg/min	↑ 2.5 ng/kg/min		↓ 2.5 ng/kg/min	↓ 5 ng/kg/min	↓ 10 ng/kg/min

*Titrate to a MAP or CPP

INOTROPES

		Below Goal	Provider specified goal	Above Goal
**Dobutamine** (CI, MAP)	**Initial Dose**	2.5 mcg/kg/min	5 minutes x 3, then 15 minutes x 2, then hourly (MAX RATE 20 mcg/kg/min)	10 minutes
	**Min Interval**	5 minutes		
	**Titrate by**	↑ 2.5 mcg/kg/min		↓ 2.5 mcg/kg/min
**Milrinone** (CI, MAP)	**Initial Dose**	0.375 mcg/kg/min*	15 minutes x 3 then hourly MAX RATE = 0.75 mcg/kg/min	15 minutes
	**Min Interval**	15 minutes		
	**Titrate by**	↑ 0.05 mcg/kg/min		↓ 0.05 mcg/kg/min

*Initial dose may be adjusted by pharmacy if CrCl <50mL/min

ANTIHYPERTENSIVES

In general, blood pressure should not be lowered by greater than 10-20% in the first hour and up to 35% over the first 24 hours unless otherwise specified by the provider.

		Above Goal	Provider specified goal	Below Goal
**Diltiazem** (BP, HR)	**Initial Bolus**	0.25 mg/kg (up to 20mg)	15 minutes x 3, then hourly (MAX RATE 15 mg/hr)	15 minutes
	**Initial Rate**	2.5 mg/hr		
	**Min Interval**	15 minutes		↓ 2.5 mg/hr
	**Titrate by**	↑ 2.5 mg/hr		
**Esmolol** (BP, HR)	**Initial Bolus**	500 mcg/kg	5 minutes x 3, 15 minutes x 2 then hourly (MAX RATE 300 mcg/kg/min)	5 minutes
	**Initial Rate**	50 mcg/kg/min		
	**Min Interval**	4 minutes		↓ 50 mcg/kg/min
	**Titrate by**	Bolus 500 mcg/kg; ↑ 50 mcg/kg/min		
**Fenoldopam** (BP)	**Initial Rate**	0.03 mcg/kg/min	15 minutes x 3 then hourly (MAX RATE 1.6 mcg/kg/min)	15 minutes
	**Min Interval**	15 minutes		
	**Titrate by**	↑ 0.05 mcg/kg/min		↓ 0.05 mcg/kg/min
**Isoproterenol** (HR)	**Initial Rate**	2 mcg/min	15 minutes x 3 then hourly (MAX RATE 10 mcg/min)	15 minutes
	**Min Interval**	10 minutes		
	**Titrate by**	↑ 1 mcg/min		↓ 1 mcg/min
**Labetolol** (BP, HR)	**Initial Bolus**	10 mg	10 minutes x 3 then hourly (MAX RATE 8 mg/min)	15 minutes
	**Initial Rate**	2 mg/min		
	**Min Interval**	10 minutes		↓ 1 mg/min
	**Titrate by**	↑ 0.5 mg/min		
**Nesiritide** (SBP)	**Initial Rate**	0.01 mcg/kg/min	Hourly (MAX RATE 0.03 mcg/kg/min)	1 hour
	**Min Interval**	3 hour		↓ 0.005 mcg/min
	**Titrate by**	↑ 0.005 mcg/kg/min		
**Nitroglycerin** (BP, Chest pain)	**Initial Rate**	10 mcg/min	5 minutes x 3, then 15 minutes x 2, then hourly (MAX RATE 400 mcg/min)	5 minutes
	**Min Interval**	3 minutes		
	**Titrate by**	Dose <20 mcg/min: ↑ 5 mcg/min<br>Dose ≥20 mcg/min: ↑10 mcg/min		↓ 10 mcg/min
**Nitroprusside** (SBP)	**Initial Rate**	0.1 mcg/kg/min	5 minutes x 3, then 15 minutes x 2, then hourly (MAX RATE 2 mcg/kg/min)	10 minutes
	**Min Interval**	3 minutes		
	**Titrate by**	↑ 0.5 mcg/kg/min		↓ 0.5 mcg/kg/min

	SBP GOAL	≥50 mmHg above goal	10-49 mmHg above goal	1-9 mmHg above goal	Provider specified goal	1-9 mmHg below goal	10-19 mmHg below goal	>20mmHg below goal
**Nicardipine** (SBP)	**Initial Rate**	7.5 mg/hr	5 mg/hr	5 mg/hr	5 minutes x 3 then every 15 minutes (MAX RATE 15 mg/hr)	10 minutes	10 minutes	5 minutes
	**Min Interval**	5 minutes						
	**Titrate by**	↑ 2.5 mg/hr	↑ 2.5 mg/hr	1st: Reassess<br>2nd: ↑ 2.5 mg/hr		↓ 2.5 mg/hr	↓ 5 mg/hr	Hold

TITRATION BY URINE OUTPUT

No Taper Required – May abruptly discontinue once infusion is no longer needed

		Below Goal	Provider specified goal	Above Goal
**Bumetanide**	**Initial Bolus**	1 mg	Hourly (MAX RATE 2 mg/hr)	30 min
	**Initial Rate**	0.5 mg/hr		
	**Min Interval**	1 hour		↓ 0.1 mg/hr
	**Titrate by**	0.25 mg/hr		
**Furosemide**	**Initial Bolus**	40 mg	Hourly (MAX RATE 60 mg/hr)	30 min
	**Initial Rate**	10 mg/hr		
	**Min Interval**	1 hour		↓ 2.5 mg/hr
	**Titrate by**	↑ 5 mg/hr		
**Vasopressin – DI**	**Initial Dose**	30 minutes	Hourly (MAX RATE 4 units/hr)	2.5 units/hr
	**Min Interval**			30 minutes
	**Titrate by**	↓ 0.5 unit/hr		↑ 0.5 unit/hr
