# Supplementary material for: The scientific rationale and study protocol for the DPP3, Angiotensin II, and Renin Kinetics in Sepsis (DARK-Sepsis) randomized controlled trial: serum biomarkers to predict response to angiotensin II versus standard-of-care vasopressor therapy in the treatment of septic shock
Source: Trials. 2024 Mar 12;25:182. doi: 10.1186/s13063-024-07995-0 (PMC10935947; doi:10.1186/s13063-024-07995-0)
Supplement: Supplementary file 4 — Additional file 4. Data collection form (DCF). [file 13063_2024_7995_MOESM4_ESM.pdf]

**Data Collection Form** (v3.1)	**Randomization Time** _______		**AT2 start time** _______	
**Study No.** ___________	**Gender:** M ☐ F ☐	**Wt:**	**BMI:** ____	**Age:** _____

	**Pre-**	**0h**	**1h**	**3h**	**6h**	**12h**	**24h**	**48h**	**72h**
**Vitals**									
MAP									
Temp									
HR									
SpO2:									
FIO2:									
**Labs**									
Art. PH:									
pCO2									
PaO2:									
serum Na									
serum K									
serum HCO3									
serum Cl									
BUN									
serum Cr									
glucose									
WBC									
Hgb									
Platelet									
T bilirubin									
Renin/DPP3	/	/	/	/	N/A		/	N/A	N/A
	24h post drug discontinuation: /								
**Pressors**									
Norepi									
Vasopressin									
AngII									
Epinephrine									
Phenyleph.									
Dobutamine									
Milrinone									
Meth. Blue									
NED									
**Other**									
P/F:									
24h UOP									
GCS:									
**SOFA**									
SOFA Resp									
SOFA Neuro									
SOFA CV									
SOFA Liver									
SOFA Coag									
SOFA Renal									
Total SOFA									

Baseline Characteristics/Comorbidities/PMHx:

ACEi exposure: Yes ☐ No ☐ ARB exposure: Yes ☐ No ☐ Diabetes: Yes ☐ No ☐
Known DM complications: Yes ☐ No ☐ (if yes, specify type): __________________ CAD/MI: Yes ☐ No ☐
CHF: Yes ☐ No ☐ (if yes, specify EF): ___________ PVD: Yes ☐ No ☐ stroke/TIA: Yes ☐ No ☐

dementia: Yes ☐ No ☐ hemiplegia: Yes ☐ No ☐ COPD: Yes ☐ No ☐ asthma: Yes ☐ No ☐

connective tissue dz: Yes ☐ No ☐ (if yes, specify type): ____________________ PUD: Yes ☐ No ☐

liver disease: Yes ☐ No ☐ cirrhosis: Yes ☐ No ☐ (if yes, specify MELD at randomization): _____________

known cirrhosis complications: Yes ☐ No ☐ (if yes, specify type): ___________________________________

CKD: Yes ☐ No ☐ (if yes, specify baseline SCr): ___ ESKD: Yes ☐ No ☐ (if yes, specify HD ☐ PD ☐ Txp ☐)

Solid tumor: Yes ☐ No ☐ (if yes, specify if metastatic Yes ☐ No ☐) Leukemia: Yes ☐ No ☐

Lymphoma: Yes ☐ No ☐ AIDS: Yes ☐ No ☐

Other Txp/immunocompromise: Yes ☐ No ☐ (if yes, specify type): _____________________________________

Vasopressors:

Norepinephrine:

Start Date: ________ Start Time: ________ End Date: ________ Duration (from start): ________

Duration (from randomization): ________ Max Dose: ________ Mean Dose: ________

Angiotensin II:

Start Date: ________ Start Time: ________ End Date: ________ Duration (from start): ________

Duration (from randomization): ________ Max Dose: ________ Mean Dose: ________

Vasopressin:

Start Date: ________ Start Time: ________ End Date: ________ Duration (from start): ________

Duration (from randomization): ________ Max Dose: ________ Mean Dose: ________

Other Adrenergic Vasopressors (doses in combined NED):

Epinephrine ☐ Phenylephrine ☐ Dopamine ☐

Start Date: ________ Start Time: ________ End Date: ________ Duration (from start): ________

Duration (from randomization): ________ Max Dose: ________ Mean Dose: ________

Inotrope Use:

Dobutamine ☐ Milrinone ☐

Start Date: ________ Start Time: ________ End Date: ________ Duration (from start): ________

Duration (from randomization): ________ Max Dose: ________ Mean Dose: ________

Start Date: ________ Start Time: ________ End Date: ________ Duration (from start): ________

Duration (from randomization): ________ Max Dose: ________ Mean Dose: ________

Methylene Blue use: yes ☐ No ☐

ICU Stay (days): ________ **Hospital Stay** (days): ________

ICU survival: Alive☐ Dead ☐ ☐**Hospital survival:** Alive☐ Death ☐

Mechanical Ventilation: Yes ☐ No ☐

Initiation date/time: ________ Stopping date/time: ________ Duration: (days)________

Re-initiation date/time: ________ Stopping date/time: ________ Duration: (days)________

Ventilator-Free Days (in first 28d post-randomization): ________

RRT: Yes ☐ No ☐

RRT type: CRRT ☐ IHD ☐ Both ☐

Initiation date/time: ________ Stopping date/time: ________ Duration (days): ________

Re-initiation date/time: ________ Stopping date/time: ________ Duration (days): ________

RRT-Free Days* (in first 28d post-randomization): ________

ARDS: Yes ☐ No ☐

AKI: Yes ☐ No ☐

Peak Serum Cr (pre-RRT): ________

AKI Stage: 1 ☐ 2 ☐ 3☐

***Adverse Events:** Yes ☐ No ☐

DVT ☐ PE ☐ arterial thrombosis ☐ (specify type): ________ atrial fibrillation ☐ tachycardia ☐

lactic acidosis ☐ limb/digit ischemia ☐ intestinal ischemia ☐ thrombocytopenia ☐ hyperglycemia ☐

confirmed infection ☐ (specify type): ________ other ☐ (specify): _____________

Notes:

*Defined as period of sustained freedom of RRT starting after last RRT; periods between RRT treatments are considered "on RRT."

†Safety events are *new* hospital-acquired events which developed *after* randomization.
